# Nile red‐based AIEgen for highly fluorescent polymer particles and its application in light‐scattering fluorescent films

**DOI:** 10.1002/smo.20240032

**Published:** 2024-09-28

**Authors:** Hongkun Jiang, Shengjie Chen, Weizhipeng Wu, Guan Wang, Xinggui Gu

**Affiliations:** ^1^ Beijing Advanced Innovation Center for Soft Matter Science and Engineering College of Materials Science and Engineering State Key Laboratory of Chemical Resource Engineering Beijing University of Chemical Technology Beijing China

**Keywords:** aggregation‐induced emission, Nile red, precipitation polymerization, solid‐state luminescence

## Abstract

Organic luminophores with superior solid‐state luminescence are urgently required in various fields, such as lighting, display, sensing, and solar energy conversion. However, to achieve their highly efficient luminescence still remains a challenge. Herein, a newly designed Nile red derivative, Nile‐DPA‐VB, is successfully obtained to exhibit aggregation‐induced emission characteristics with the photoluminescent quantum yield (PLQY) of 11.45%. Such PLQY could be further promoted to 53.45% when Nile‐DPA‐VB is polymerized undergoing precipitation polymerization process, where the confined aggregation microenvironment severely restricts the intramolecular motions of Nile‐DPA‐VB. Remarkably, Nile‐DPA‐VB is ultrasensitive to the polarity and steric effect, enabling the real‐time monitoring of aggregation microenvironment evolution for precipitation polymerization. The microphase separation and dynamic hardening for the nucleation and growth processes are visually demonstrated, which contribute dominantly to the high‐efficiency luminescence. Finally, by doping the as‐prepared fluorescent polymeric particles into polymethyl methacrylate, functional films with high luminescence and high haze are achieved to show the potential in lighting. These findings clearly demonstrate the significant role of polymerization in constructing high‐efficiency solid‐state luminescent materials for practice.

## Graphical abstract

Based on Nile red, an AIEgen of Nile‐DPA‐VB was synthesized, exhibiting the high‐efficiency solid‐state luminescence. The luminescence was further promoted by aggregation microenvironment manipulation through taking Nile‐DPA‐VB as the monomer for precipitation polymerization. Thus, highly fluorescent polymer particles based on the AIE‐active Nile red derivative were harvested, demonstrating their promising application in functional film.

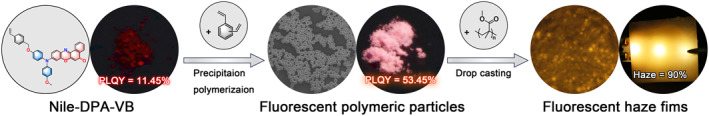

## 1 INTRODUCTION

With the merits of structural multiplicity and tunability, organic luminogens, such as Nile red, have garnered increasing attention as a promising candidate in lighting, displays, sensing, energy utilization, and so on.[^1^, ^2^, ^3^, ^4^] High luminescence efficiency of organic luminogens crucially determined the practical performance and thus was extensively pursued during these applications. However, conventional organic luminogens, typically with planar and conjugated structures, always suffered from emission quenching in the concentrated solutions or solid due to luminescence‐detrimental π‐π stacking with the formation of excimers,^[5]^ known as aggregation caused quenching (ACQ),^[6]^ which seriously limits their application in the aggregated state. The concept of aggregation‐induced emission (AIE) coined by Tang et al. effectively overcame the limitations of ACQ and opened new avenues for achieving highly effective luminogens.^[7]^ Featured with propeller‐like structures, luminogens with AIE features (AIEgens) suppressed the detrimental π‐π stacking through the steric hindrance for high‐efficiency solid‐state luminescence, and earned flourishing development.[^8^, ^9^, ^10^] However, the flexible molecular conformation of AIEgens intrinsically brought about a loose aggregation microenvironment, which still provided them with the space for molecular motions and consequently weakened their luminescence efficiency.[^11^, ^12^, ^13^]

Various strategies have been developed for prompting the performance of AIEgens in the aggregated state.[^14^, ^15^, ^16^, ^17^] Among them, crystal engineering is the primary strategy to improve the emission of AIEgens in solids. As reported by Liu et al.,^[18]^ nanocrystals of AIEgens fabricated from disordered nanoaggregates using ultrasonication demonstrated the increased photoluminescent quantum yield (PLQY). By promoting intermolecular interactions and electron coupling, alkoxy spacers enable brighter and longer‐lasting phosphorescence. The formation of a highly ordered nanocrystalline lattice restricts intramolecular rotations and vibrations, reducing non‐radiative decay pathways. The closer packing of molecules within the lattice facilitates efficient energy transfer, further improving the PLQY. Apart from the crystal engineering, the confined aggregation microenvironment could also be manipulated by the self‐assembly strategy.[^11^, ^15^] Weak noncovalent interactions between the polymer matrix and AIEgens restricted intramolecular motions and promoted luminescence enhancement, which minimized the non‐radiative decay. As a result, the excited states were stabilized, leading to the enhanced PLQY. In our previous work, by employing the corannulene‐decorated PEG as the polymer matrix, a fourfold increase in PLQY compared to that with DSPE‐PEG as the polymer matrix was achieved, highlighting the restricted intramolecular motions through the host–guest interactions.^[19]^ This strategy has been explored in further work reported by Hu et al., with examples demonstrating the improved fluorescence in AIE nanoparticles using polystyrene (PS)‐modified PEG^[20]^ and NIR‐emitting AIEgens‐loaded PS nanospheres.^[21]^ However, the unstable construction of the self‐assemblies obtained from the non‐covalent interactions as well as the complex synthesis produces limited further improvement in luminescence performance, and the development of an advantageous strategy remains a challenge.

**FIGURE 1:**
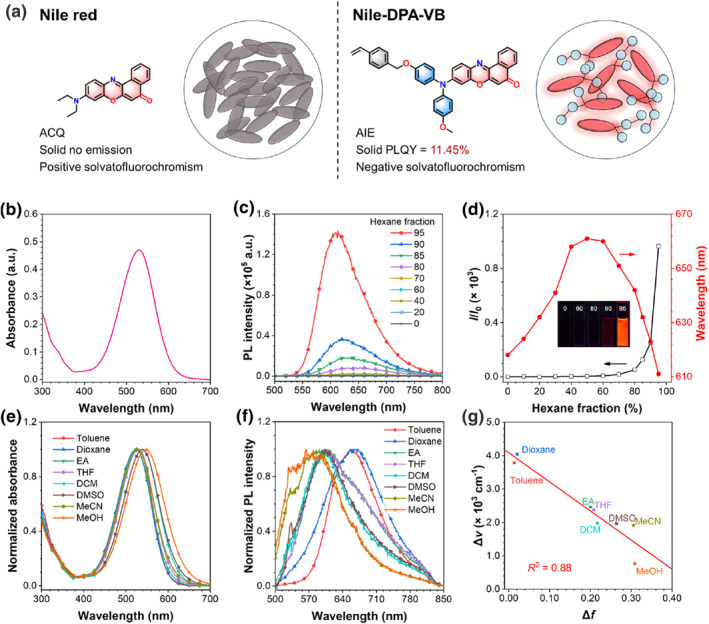
(a) Chemical structures and schematic illustration of the solid‐state luminous behavior of Nile red and Nile‐DPA‐VB. (b) Absorption spectrum of Nile‐DPA‐VB (1 × 10^−5^ M) in THF solution. (c) PL spectra of Nile‐DPA‐VB (1 × 10^−5^ M) in THF/hexane solutions with different hexane fractions. *λ*_ex_ = 460 nm. (d) The plot of *I*/*I*_0_ and maximum emission wavelength versus hexane fraction for Nile‐DPA‐VB (1 × 10^−5^ M) in THF/hexane solutions, where *I* represents the maximum PL intensity of THF/hexane solution; *I*_0_ represents that in pure THF solution. Inset: Photographs of Nile‐DPA‐VB THF/hexane solutions with hexane fractions of 0%, 60%, 80%, 90%, and 95% under a 365 nm UV irradiation. (e) Normalized absorption and (f) PL spectra of Nile‐DPA‐VB (1 × 10^−5^ M) in different solvents. *λ*_ex_ = 460 nm. (g) Lippert–Mataga plot of Nile‐DPA‐VB in different solvents. PL, photoluminescence; THF, tetrahydrofuran.

In this study, by synthesizing polymerizable AIEgens based on the Nile red, we reported an effective strategy to promote luminescence efficiency based on the confined aggregation microenvironment established via precipitation polymerization. An AIE‐active monomer, Nile‐DPA‐VB, was synthesized by modifying the traditional ACQ luminogens of Nile red with twisted benzene rings, achieving a solid‐state PLQY of 11.45% (Figure 1a). Such PLQY of Nile‐DPA‐VB was further promoted to 53.45% by polymerizing Nile‐DPA‐VB with divinylbenzene (DVB) in acetonitrile. Interestingly, Nile‐DPA‐VB exhibited the distinct AIE features and unique negative solvatofluorochromic effect, endowing it with the ultrasensitivity to monitor the formation of fluorescent polymeric particles (FPPs) through precipitation polymerization and reveal the underlying mechanism of the polymerization enhanced emission. As a result, the nucleation with microphase separation was identified and the dynamic hardening in precipitation polymerization for suppressing intramolecular motions by the steric effect of the highly cross‐linked polystyrene matrix was revealed. Finally, uniform FPPs with controllable sizes were harvested and their potential in functional luminescent films with effective luminescence and high haze were demonstrated.

## 2 RESULTS AND DISCUSSION

### 2.1 Synthesis and characterization of Nile‐DPA‐VB

AIE‐active Nile‐DPA‐VB was synthesized through the routes shown in Figure S1, where a twisted diphenylamine donor was introduced to reduce the face‐to‐face stacking in aggregation and the external vinylbenzyl group was attached as an active site moiety for polymerization. The synthetic routes and structural characterizations including ^1^H and ^13^C nuclear magnetic resonance spectroscopy and high resolution mass spectrometry are provided in Figures S2–S8. The photophysical properties of the Nile‐DPA‐VB were measured in tetrahydrofuran (THF) solution. As shown in Figure 1b, Nile‐DPA‐VB featured with a primary absorption band at 530 nm with a molar extinction coefficient of 4.70 × 10^4^ M^−1^ cm^−1^, which is higher than that of Nile red.^[22]^

Aggregation‐induced emission characteristics of the Nile‐DPA‐VB were determined by adding the poor solvent of hexane into the Nile‐DPA‐VB solution with THF as the good solvent, and the results are shown in Figure 1c,d. As Nile‐DPA‐VB was dissolved in THF, weak fluorescence was detected. While, with the addition of hexane, the fluorescence intensities were maintained slightly and it enhanced dramatically as hexane fraction over 80%. The PL intensity at 95% hexane content increased, nearly 1000 times compared to that in pure THF solution, indicating that Nile‐DPA‐VB exhibits obvious AIE characteristics. Besides, the PLQY of Nile‐DPA‐VB solid powder was evaluated to 11.45%, whereas that of Nile red was nearly 0%.^[6]^

Interestingly, the emission peaks in the THF/hexane solution were first redshifted and then blueshifted with the addition of hexane, revealing a complicated response to the surrounding microenvironment. In order to reveal that the effects of polarity on the absorption and PL spectra of Nile‐DPA‐VB were characterized. As shown in Figure 1e,f, with the solvent polarity increased, the absorption peaks of Nile‐DPA‐VB generally underwent a redshift from 528 nm in toluene to 548 nm in methanol. Correspondingly, a blueshift in the emission peaks was also detected, shifting from 660 nm in toluene to 572 nm in methanol. Based on this, the Lippert‐Mataga plot was calculated in Figure 1f, indicating the negative solvatofluorochromism of Nile‐DPA‐VB.^[23]^ Different from the typical AIEgens with the twisted intramolecular charge transfer effect, emissions of Nile‐DPA‐VB exhibited a negative correlation to the polarity of solvents. The reasons for these interesting results could be assigned to the molecular skeleton with carbonyl and heteroatoms, which promotes a stable zwitterionic form in polar environments.^[24]^ Therefore, with the modification of the typical luminogens of Nile red, AIE‐active luminogens with high‐efficiency solid‐state luminescence were achieved and could be employed as the monomer for precipitation polymerization.

### 2.2 Precipitation polymerization of Nile‐DPA‐VB

**FIGURE 2:**
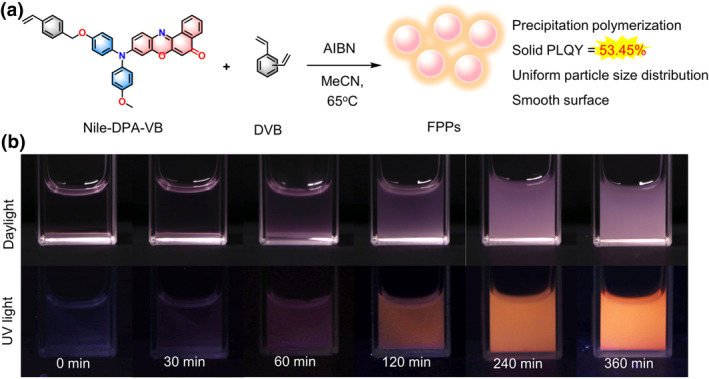
(a) Schemic illustration of precipitation polymerization using Nile‐DPA‐VB and DVB as monomers and AIBN as the initiator in acetonitrile (MeCN) at 65°C. (b) Photographs of polymerization proceeded under daylight and 365 nm UV light at the scheduled time. AIBN, azobisiobutyronitrile; DVB, divinylbenzene.

Precipitation polymerization of Nile‐DPA‐VB was conducted under a nitrogen atmosphere in acetonitrile at 65°C, employing a mass ratio of 1:1000:20 for the monomers Nile‐DPA‐VB and DVB, along with the initiator azobisisobutyronitrile (AIBN). With the polymerization proceeding, the solution was initially transparent and progressively became turbid, indicative of the occurrence of precipitation polymerization, as shown in Figure 2b. At primary 30 min, the reaction mixture remained a clear purple‐red color, while as the polymerization proceeded to 60 min, the turbidity became discernible. Finally, the reaction solution transformed into a completely opaque pink suspension at 360 min. Intriguingly, the emission under UV irradiation also exhibited a distinct variation, where the fluorescence was negligible at 0 min and it enhanced with an orange‐red color as the polymerization prolonged to 360 min. Considering the polymerization reactivity of Nile‐DPA‐VB and its AIE properties, it could be inferred that the Nile‐DPA‐VB have been polymerized into the obtained FPPs.

**FIGURE 3:**
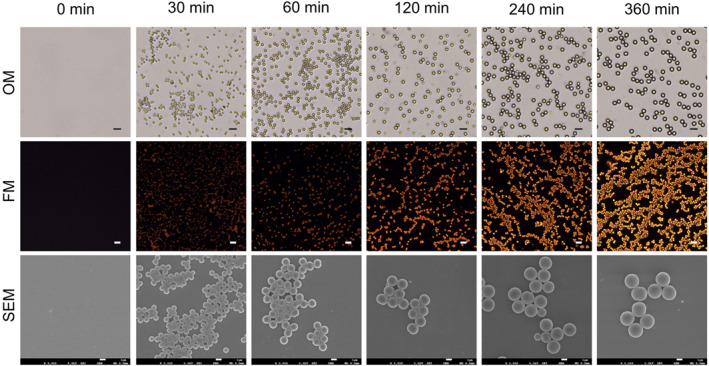
OM, FM, and SEM images of fluorescent polymeric particles captured at the scheduled reaction time. Scale bars: OM images 10 μm, FM images 10 μm, SEM images 1 μm. OM, optical microscopy; FM, fluorescent microscopy; SEM, scanning electron microscopy.

To confirm the formation of FPPs, the precipitation polymerization was further investigated by optical microscopy (OM), fluorescent microscopy (FM), and scanning electron microscopy (SEM) (Figures 3, S9 and S10). As shown in the OM images in Figure 3, no obvious objects were observed at 0 min, while particles with spherical morphology appeared at 30 min and progressively increased in size from 30 to 360 min. Similarly, as shown in the FM images, tiny fluorescent dots could be detectable at 30 min, which became more distinct and brighter as time passed. At 360 min, a multitude of orange light particles were detected. Based on this, it could conclude that FPPs were formatted from the polymerization, and the fluorescence of the reaction solutions was driven from the FPPs, which suggested that Nile‐DPA‐VB molecules were polymerized into FPPs.

Besides, the formation of FPPs was further characterized by SEM. In Figures 3 and S9, it could be found that no particles were detected before 9 min, while spherical particles emerged after 15 min. As illustrated in Figure 3, the spheres grew progressively from 30 to 360 min, maintaining a uniform size. Therefore, quantifications of the particle size and distribution were conducted with the SEM images subjected to ImageJ software,^[25]^ and the results are shown in Tables S3 and S4 and Figures S11–S13. From the results, the particle size was calculated with 0.916 μm at 30 min, it further expanded to 1.656 μm at 90 min, and then it grew to 2.434 μm at 360 min. Throughout the process, the dispersion coefficients remained below 1.050, which met the criterion of monodisperse,^[26]^ indicating a homogeneous distribution suitable for further processing.

With the ultrasensitivity of Nile‐DPA‐VB to the aggregation microenvironment on aspects of steric effect and polarity derived from the AIE features and solvatofluorochromic effect, it could be employed as the ultrasensitive probe to monitor the processes of precipitation polymerization. Generally, precipitation polymerization is conceptualized as a two‐stage process comprising nucleation and growth processes.[^27^, ^28^] During the nucleation stage, the initiator is activated to produce free radicals and initiate the polymerization of the monomer. As the polymer chains propagate, it would precipitate and aggregate into the primary nuclei. Growth processes proceeded with monomers or short polymer chains deposited onto the surfaces of nuclei, leading to an increase in particle sizes. The mechanism encompasses complicated molecular chain aggregation behaviors and aggregation microenvironment alternations, which made it difficult to monitor by the traditional methods, such as SEM, transmission electron microscope (TEM), and so on.^[29]^ Therefore, based on the ultrasensitivity of Nile‐DPA‐VB to the steric effect and polarity, monitoring of the precipitation polymerization mechanism could be achieved in a real‐time manner.

**FIGURE 4:**
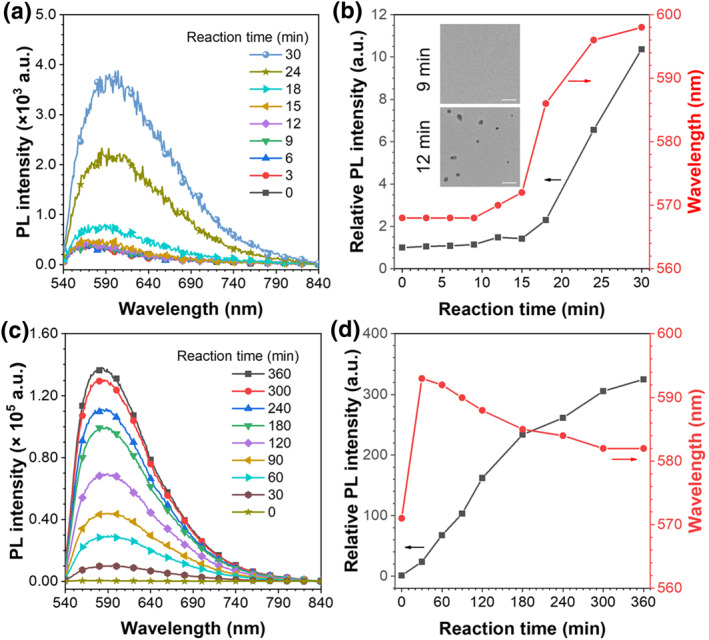
(a) PL spectra, (b) changes in relative PL intensity and maximum emission wavelength in the initial 30 min *λ*_ex_ = 460 nm. Inset: Transmission electron microscope images of fluorescent polymeric particles at 9 and 12 min. Scale bar: 200 nm. (c) PL spectra, (d) changes in relative PL intensity and maximum emission wavelength in the whole process. *λ*_ex_ = 460 nm. PL, photoluminescence.

PL spectra during the polymerization were conducted to elucidate the aggregation microenvironment evolution for particle formation. As shown in Figure 4a,b, the emission of the reaction solution persisted unchanged within 9 min, and a subtle redshift as well as a slight enhancement at 12 min. As the polymerization proceeded to 30 min, the emission redshifted dramatically from 568 to 598 nm, and about 10‐fold enhancement in PL intensities was detected. Based on the merits of Nile‐DPA‐VB, it could be inferred that strong polarity alterations and weak aggregation changes could be anticipated during the process. Furthermore, TEM was employed to verify the morphology changes of the reaction solutions. As shown in Figure 4b, no particles were found at 9 min, whereas irregular dots emerged at 12 min, demonstrating the occurrence of the nucleation. Collectively, the mechanism of nucleation could be sensitively monitored with the polymer chains aggregation and polarity alternations. As the polymerization was initiated by AIBN at the primary 9 min, the obvious introducing period was detected with negligible emission changes in intensities and emission peaks. Meanwhile, the precipitation of the polymer chain and the formation of the nuclei from 12 to 30 min resulted in the resistance of the intramolecular motions of Nile‐DPA‐VB, which caused the enhancement in PL intensities. Additionally, monomers of the DVB were absorbed into the nuclei for further polymerization, leading to the microphase separation of the nuclei from the reaction solvents and the dramatic redshift in its emission peaks. Therefore, the subtle nucleation of precipitation polymerization was distinguished by the ultrasensitive Nile‐NPA‐VB on the aspects of polymer chain aggregation and polarity alternations.

The growth processes proceeded following the nucleation, which could also be investigated by PL spectra. In this stage, polymerization proceeded with the increase in PL intensities. Interestingly, as the distinct comparison with nucleation processes, an obvious blueshift was detected in this process, as shown in Figure 4d. As reported in our previous work,^[30]^ dynamic hardening was recognized and dominated the polymer chain aggregation in growth processes. Dynamic hardening is a specific process similar to crystallization. In this process, the newly formed polymer chains successively aggregated and collapsed on the surface of the as‐formed polymeric particles, accompanying with the solvent extrusion, which caused the significant enhancement in aggregation confinement of polymer chains and it would be further boosted by introduction of crosslinkers.^[31]^ In this work, the crosslinker of DVB was used as the dominating monomer for precipitation polymerization, which would extremely aggravate the aggregation of the polymer chains and harvest the highly crosslinked FPPs. Therefore, the dynamic hardening process of the growth processes suppressed the intramolecular motions of Nile‐DPA‐VB in polymer chains and disrupted the planarity of the Nile‐DPA‐VB, leading to a significant increase in PL intensity and a blue shift in emission peak. As a result, the PLQYs of the FPP powder continued to increase with particle size, as listed in Table 1. The particles with sizes of 0.916 and 2.434 μm exhibited PLQYs of 29.48% and 53.45% (Table 1), respectively, which were nearly two and four times higher than that of the Nile‐DPA‐VB powder, demonstrating that polymerization is an effective strategy for enhancing the solid‐state luminescence.

**TABLE 1:** PLQYs of fluorescent polymeric particles with different sizes.

*D*_n_ (μm)	PLQY (%)
0.916	29.48
1.350	39.13
1.783	41.40
2.193	49.03
2.434	53.45

Abbreviation: PLQY, photoluminescent quantum yield.

### 2.3 Preparation and characterization of haze fluorescent films

**FIGURE 5:**
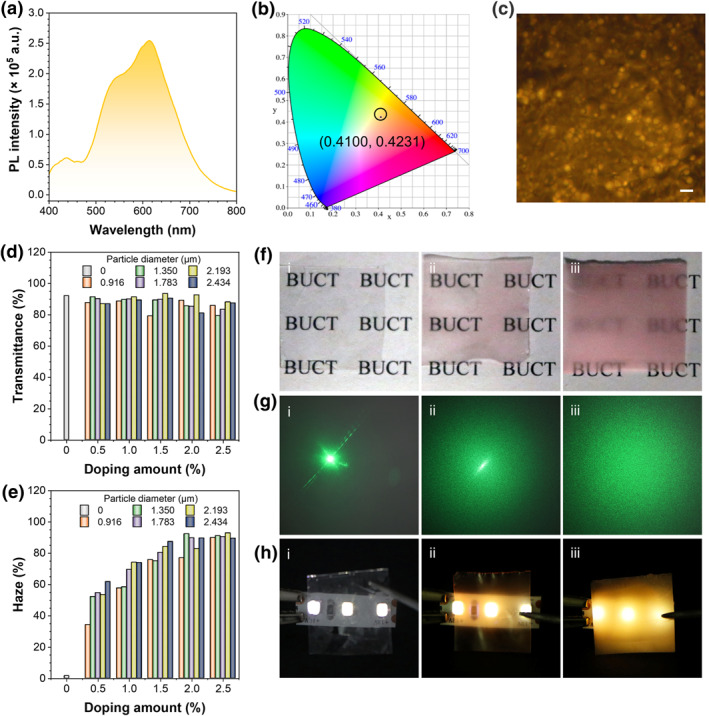
(a) PL spectra, (b) CIE 1931 chromaticity diagram, and (c) fluorescent microscopy image of FPPs into PMMA film doped with 2.434 μm FPPs at the mass ration of 2.5%. *λ*_ex_ = 365 nm. Scale bar: 5 μm. (d) Transmittance and (e) haze histogram of PMMA films doped with FPPs of different mass fractions and particle sizes. (f) Photographs of the films placed on “BUCT” pattern under daylight. (g) Photographs of the light spots created by a 100 mW 532 nm laser pointer through the films. (h) Photographs of the films above a 365 nm UV LED strip. Doping mass fraction of 2.434 μm FPPs: film i, 0%; film ii, 0.5%; film iii, 2.5%. FPP, fluorescent polymeric particle; PL, photoluminescence; PMMA, polymethyl methacrylate.

With the high efficiency solid‐state luminescence and uniform size of FPPs (Figure S14), the potential in the luminescent film was demonstrated. Here, the fluorescent film was fabricated by dropping FPPs into polymethyl methacrylate (PMMA). As shown in Figure 5a,b, yellow emission with the CIE coordination of (0.410, 0.423) for FPPs‐doped PMMA film was achieved with a PLQY up to 64.58%. FM shown in Figure 5c and cross‐section SEM images in Figure S15 revealed that FPPs were well‐distributed into the film. However, the highly crosslinked FPPs comprised by DVB exhibited incompatibility in PMMA, leading to the difference in light transmission and solid‐solid interface, which made it the candidate for light scattering.[^32^, ^33^]

As such, the transmittance and haze of the FPPs doped PMMA films were characterized (Figures S16–S21), and the effects of size and doping concentration on haze were determined in Figure 5d,e. From the results, the incorporation of FPPs barely affected the transmittance in the visible range compared to pure PMMA films. All films showed an average transmittance of more than 80%, with no significant correlation with particle size or doping amount of the FPPs. In contrast, the haze was positively correlated with the particle size enlarged and the amount of FPPs increased. At a 0.5% doping mass fraction, films containing 0.916 μm FPPs exhibited less than 35% haze, and those with 2.434 μm FPPs showed more than 60% haze; whereas when the doping amount reached 2.5%, the haze of all films containing FPPs was over 85%. By optimizing the doping formulation, films with FPPs could be characterized by high fluorescence, transmittance, and haze simultaneously.

In particular, three films were selected to visually demonstrate the effect of haze: the first one of pure PMMA film (film i), the second one with 0.5% mass fraction of 2.434 μm FPPs (film ii), and the third one with 2.5% doping amount of the same particles (film iii). Letter patterns, a 532 nm green laser pointer, and a 365 nm UV strip were used to demonstrate the scattering ability of the films for daylight, linear light, and their own fluorescence, respectively. Photographs are presented in Figure 5f,h. The BUCT letters were legible through films i and ii while blurred under film iii; the laser pointer generated a distinct dot through film i, a dot with speckles through film ii, and only speckles through film iii; when overlayed on the UV strip, film i showed no fluorescence, film ii exhibited partial fluorescence, while film iii glowed uniformly. The film with a 2.5% doping fraction demonstrated effective light scattering across all conditions and could be a promising candidate for use as a soft luminescent material.

## 3 CONCLUSION

In summary, by modifying the typical dye of Nile red, high‐efficiency solid‐state luminescent material of Nile‐DPA‐VB was achieved, and the PLQY was further boosted through the strategy based on aggregation microenvironment manipulation via precipitation polymerization. By integrating molecular rotation and steric hindrance, AIE‐active Nile‐DPA‐VB was synthesized, which exhibited solid‐state emission with a PLQY of 11.45%. The PLQY of FPPs was further promoted to 53.45% by constructing the confined aggregation microenvironment via precipitation polymerization, exhibiting nearly four times enhancement compared to that of Nile‐DPA‐VB powder. Additionally, taking the merits of multi‐rotor structure and donor–acceptor effect into account, Nile‐DPA‐VB bears with the ultrasensitivity to the aggregation microenvironment on the aspects of polarity and steric effect. Based on this, monitoring of the precipitation polymerization process was achieved, which revealed the microphase separation during the nucleation stage and the dynamic hardening in the growth stage, extending the understanding of precipitation polymerization and obtaining efficiently luminescent FPPs. Besides, taking advantage of the high‐efficiency solid‐state luminescence, FPPs were employed to fabricate highly efficient luminescent films, and luminescent films with controllable haze adjusted through doping were achieved. This work provides an efficient strategy for achieving high‐efficiency solid‐state luminescent materials, which opens up new possibilities for constructing lighting materials.

## CONFLICT OF INTEREST STATEMENT

The authors declare no conflicts of interest.

## ETHICS STATEMENT

No animal or human experiments were involved in this study is sufficient.

## Supporting information

Supporting Information S1

## Data Availability

The data that support the findings of this study are available from the corresponding author upon reasonable request.
