# Supplementary material for: Nile red‐based AIEgen for highly fluorescent polymer particles and its application in light‐scattering fluorescent films
Source: Smart Mol. 2024 Sep 28;3(1):e20240032. doi: 10.1002/smo.20240032 (PMC12117904; doi:10.1002/smo.20240032)
Supplement: Supplementary file 1 — Supporting Information S1 [file SMO2-3-e20240032-s001.docx]

Supporting Information

Nile Red-Based AIEgen for Highly Fluorescent Polymer Particles and Its Application in Light-scattering Fluorescent Films

*Hongkun Jiang^#^, Shengjie Chen^#^, Weizhipeng Wu, Guan Wang* and Xinggui Gu**

**Materials**

4-bromophenol, 4-vinylbenzyl chloride, 2-chloro-1,4-naphthoquinone, 2-amino-5-bromophenol, divinylbenzene (80% isomeric mixture, DVB-80), 2,2'-azobis(2-methylpropionitrile) (AIBN), and polymethyl methacrylate (PMMA, average Mw = 100,000) were purchased from Energy-Chemical. P-Anisidine, tris(dibenzylideneacetone)dipalladium, tri-tert-butylphosphine, tri-tert-butylphosphonium tetrafluoroborate, sodium tert-butoxide, deuterochloroform (CDCl_3_), and dimethyl sulfoxide-d_6_ (DMSO-d_6_) was purchased from Inno-Chem. Purified water was purchased from Wahaha. Dichloromethane (DCM), petroleum ether (PE), toluene, tetrahydrofuran (THF), Ethyl acetate (EA), acetonitrile (MeCN), hexane, potassium carbonate (K_2_CO_3_), anhydrous sodium sulfate (Na_2_SO_4_) and sodium chloride (NaCl) were purchased from Beijing Chemical Group. DVB-80 was purified by vacuum distillation. AIBN was recrystallized in methanol before using. Solvents including DCM, PE, toluene and THF were distilled. Other agents and solvents were used as received without further purification.

**Instruments**

^1^H and ^13^C spectra were measured on Bruker 400 M NMR spectrometers. High-resolution mass spectra (HRMS) were recorded on a Bruker BIFLEX III mass spectrometer system. UV-Vis absorption spectra were taken on a Shimadzu UV-2600 spectrophotometer. Photoluminescence (PL) spectra were measured using an Edinburgh FS5 fluorescence spectrophotometer. Photoluminescence quantum yields (PLQY) were measured using a FLS980 fluorescence spectrophotometer. Total and diffuse transmission spectra were taken using a Shimadzu UV-3600i spectrophotometer. Optical microscope (OM) and fluorescent microscope (FM) images were captured on an OLYMPUS BX53M microscope. Scanning electron microscopy (SEM) images were captured using a JEOL JMS-7800F schottky field emission scanning electron microscope. Transmission electron microscopy (TEM) images were captured using a Hitachi HT7700 120 kV transmission electron microscope. Photographs of solution and powder were taken using a Canon EOS 80D camera.

**Synthesis**

**Figure S1.** Synthetic routes of Nile-DPA-VB.

**Synthesis of Compound 1**

2-amino-5-bromophenol (1.5039 g, 7.998 mmol), 2-chloro-1,4-naphthoquinone (1.5419 g, 8.006 mmol) and K_2_CO_3_ (1.1563 g, 8.366 mmol) were added to dry acetonitrile (100 mL) under N_2_ atmosphere. The mixture was heated to 80 ^o^C and stirred overnight to give a dark-yellow suspension. After cooling to room temperature, the mixture was filtered, washed with methanol and water, and then dried under vacuum to obtain a yellow solid (2.3189 g, 6.558 mmol) with an 82.0% yield. ^1^H NMR (400 MHz, CDCl_3_), *δ* (ppm): 8.72-8.67 (m, 1H), 8.32-8.26 (m, 1H), 7.82-7.74 (m, 2H), 7.69 (d, 1H, *J* = 8.0 Hz), 7.52-7.46 (m, 2H), 6.45 (s, 1H). ^13^C NMR (100 MHz, CDCl_3_), *δ* (ppm): 183.9, 150.6, 147.7, 144.3, 132.2, 132.1, 131.9, 131.1, 130.8, 128.7, 126.1, 124.8, 119.3, 107.9.

Compound **2** and **3** was synthesized according to the literature.^[1]^

**Synthesis of Nile-DPA-VB**

Compound **1** (0.5912 g, 1.813 mmol), Compound **3** (0.7729 g, 2.332 mmol), tris(dibenzylideneacetone)dipalladium (0.0771 g, 0.084 mmol), tri-tert-butylphosphine tetrafluoroborate (0.0618 g, 0.213 mmol) and sodium tert-butoxide (0.3206 g, 3.336 mmol) were added to dry toluene (40 mL) under N_2_ atmosphere. The mixture was heated to 110 ^o^C and stirred for 9 h, resulting in a purplish-black liquid. After evaporation of solvent, the residue was extracted and washed with ethyl acetate for three times. The crude product was purified by column chromatography (PE/EA = 5/1, v/v) to obtain a blank solid (0.8508 g, 1.475 mmol) with an 81.4% yield. ^1^H NMR (400 MHz, CDCl_3_), *δ* (ppm): 8.67 (d, 2H, *J* = 8.0 Hz), 8.30 (d, 2H, *J* = 8.0 Hz), 7.74 (t, 1H, *J* = 8.0 Hz), 7.68 (t, 1H, *J* = 8.0 Hz), 7.56 (d, 2H, *J* = 8.0 Hz), 7.45 (d, 2H, *J* = 8.0 Hz), 7.40 (d, 2H, *J* = 8.0 Hz), 7.16 (d, 2H, *J* = 8.0 Hz), 7.00 (d, 2H, *J* = 8.0 Hz), 6.93 (d, 2H, *J* = 8.0 Hz), 6.83 (d, 2H, *J* = 8.0 Hz), 6.77 (dd, 1H, *J* = 20.0, 8.0 Hz), 6.66 (s, 1H), 6.39 (s, 1H), 5.77 (d, 1H, *J* = 20.0 Hz), 5.28 (d, 1H, *J* = 12.0 Hz), 5.06 (s, 2H), 3.83 (s, 3H). ^13^C NMR (100 MHz, CDCl_3_), *δ* (ppm): 183.9, 157.6, 156.7, 152.1, 151.8, 145.8, 142.1, 138.8, 138.5, 137.5, 136.4, 136.2, 131.9, 131.8, 131.5, 130.5, 130.4, 128.1, 128.0, 127.7, 127.0, 126.5, 125.8, 124.0, 116.1, 115.2, 114.3, 106.2, 102.9, 70.1, 55.5. HRMS (MALDI-TOF, m/z): [M]^+^ calcd for C_38_H_28_N_2_O_4_, 576.2049; found, 576.2044.

**Calculations of Lippert-Mataga equation^[2]^**

The Lippert-Mataga plot of Nile-DPA-VB was constructed by using the relation:

(1)

$$\Delta v=v_{A}-v_{F}=\frac{2\Delta\left( \mu_{e}-\mu_{g} \right)^{2}}{hca^{3}}\Delta f+\mathrm{const}$$

where $\Delta v$ is the Stokes shift (expressed as wavenumber) between the absorption and PL emission peaks, $\mu_{e}$ and $\mu_{g}$ are the dipole moments in the ground and excited states, respectively, $h$ is the Planck constant, $c$ is the speed of light in vacuum, $a$ is onsager cavity radius.

The orientation polarizability of the solvents $\Delta f$ is defined as:

(2)

$$\Delta f=\frac{\varepsilon-1}{2\varepsilon+1}-\frac{n^{2}-1}{2n^{2}+1}$$

where $\varepsilon$ and $n$ are the dielectric constant and refractive index of the solvents, respectively.

**Table S1** Orientation polarizability values of various solvents.

| Solvents | $\varepsilon$ | $n$ | $\Delta f$ |
| --- | --- | --- | --- |
| Toluene | 2.38 | 1.496 | 0.014 |
| Dioxane | 2.21 | 1.422 | 0.021 |
| EA | 6.03 | 1.372 | 0.200 |
| THF | 7.39 | 1.404 | 0.208 |
| DCM | 8.9 | 1.424 | 0.217 |
| DMSO | 48.9 | 1.479 | 0.264 |
| MeCN | 37.5 | 1.344 | 0.305 |
| MeOH | 32.6 | 1.328 | 0.309 |

**Table S2** Stokes shift values of Nile-DPA-VB in various solvents.

| Solvents | $\lambda_{A}$ (nm) | $\lambda_{F}$ (nm) | $v_{A}$ (cm^-1^) | $v_{F}$ (cm^-1^) | $\Delta v$ (cm^-1^) |
| --- | --- | --- | --- | --- | --- |
| Toluene | 528 | 660 | 18939 | 15152 | 3787 |
| Dioxane | 524 | 665 | 19084 | 15038 | 4046 |
| EA | 525 | 603 | 19048 | 16584 | 2464 |
| THF | 529 | 605 | 18904 | 16529 | 2375 |
| DCM | 538 | 602 | 18587 | 16611 | 1976 |
| DMSO | 539 | 603 | 18553 | 16584 | 1969 |
| MeCN | 527 | 586 | 18975 | 17065 | 1910 |
| MeOH | 548 | 572 | 18248 | 17483 | 765 |

**Preparation and sampling of fluorescent polymer particles (FPPs)**

Fluorescent polymer particles (FPPs) were prepared by precipitation polymerization. In a typical reaction, Nile-DPA-VB (1.10 mg, 0.00191 mmol, 0.1% w/w of monomer), AIBN (21.96 mg, 0.134 mmol, 2% w/w of monomer), and DVB-80 (1.2 mL, 8.425 mmol, 3% v/v of solvent) were fully dissolved in 40 mL of acetonitrile using ultrasonication. After purging with nitrogen for 20 minutes, the mixture was placed in an oil bath preheated to 65°C and then heated for 6 hours, with magnet stirring at 50 rpm.

Samples were extracted using a syringe at different reaction times and immediately quenched by ice water. To monitor the initial stage, samples were taken at 3-minute intervals for the first 30 minutes. To monitor the overall reaction, samples were collected at 30-minute intervals during the first 2 hours and then at 60-minute intervals until 6 hours.

The solid powder of FPPs is obtained by centrifugation.

**Optical Microscope (OM) measurement**

Samples were washed with EA, dropped onto a glass slide and covered with a coverslip. Images were taken under bottom-up white light.

**Fluorescent Microscope (FM) measurement**

Samples were dropped onto a glass slide directly and left to stand in air until the solvent was completely evaporated. Images were taken under 365 nm UV irradiation.

**SEM and TEM measurements**

Samples were diluted with acetonitrile and dropped onto the silicon slice and copper mesh for SEM and TEM measurements, respectively.

**Statistics of particle size distribution**

200 particles were selected and manually measured using ImageJ software^[3]^ based on each SEM image. The average particle size and distribution coefficient are calculated as follows:

Linear average diameter

(3)

$$D_{n}=\sum_{i=1}^{n} {D_{i}}/n$$

Weight average diameter

(4)

$$D_{w}={\sum_{i=1}^{n} D_{i}^{4}}/{\sum_{i=1}^{n} D_{i}^{3}}$$

Polydispersity

(5)

$$U={D_{w}}/{D_{n}}$$

**Table S3** Particle size distribution of FPPs at different reaction time in the early stage.

| Reaction time(min) | D_n_ (μm) | D_w_ (μm) | U |
| --- | --- | --- | --- |
| 0 | 0.0 | 0.0 | / |
| 9 | 0.0 | 0.0 | / |
| 12 | 28.7 | 40.0 | 1.395 |
| 15 | 322.6 | 359.4 | 1.114 |
| 18 | 516.8 | 542.9 | 1.050 |
| 24 | 684.8 | 716.3 | 1.046 |
| 30 | 811.5 | 836.3 | 1.031 |

**Table S4** Particle size distribution of FPPs at different reaction time.

| Reaction time(min) | D_n_ (μm) | D_w_ (μm) | U |
| --- | --- | --- | --- |
| 30 | 0.916 | 0.929 | 1.015 |
| 60 | 1.350 | 1.376 | 1.020 |
| 90 | 1.621 | 1.656 | 1.021 |
| 120 | 1.783 | 1.834 | 1.028 |
| 180 | 2.032 | 2.081 | 1.024 |
| 240 | 2.193 | 2.272 | 1.036 |
| 300 | 2.369 | 2.419 | 1.021 |
| 360 | 2.434 | 2.489 | 1.022 |

**Preparation of fluorescent haze films**

Preparation of fluorescent haze films doped with FPPs: 100 mg of PMMA was dissolved in 2 mL of DCM. FPPs samples with different diameters (0.916, 1.350, 1.783, 2.193, and 2.434 μm) and amounts (0.5, 1.0, 1.5, 2.0, and 2.5 mg) were added, respectively. Each of the mixture was mixed uniformly under ultrasonication, and then dropped on a 75 × 25 × 1 mm glass slide using a syringe. The films were demolded after the solvent fully evaporated and dried at room temperature for 12 hours.

Preparation of blank control pure PMMA film: 100 mg of PMMA was dissolved in 2 mL of DCM and dropped on a 75 × 25 × 1 mm glass slide using a syringe. The film was demolded after the solvent fully evaporated and dried at room temperature for 12 hours.

**Calculation of transmittance and haze:**

The data of full transmission and diffuse transmission spectrum of films were used to calculate the transmittance and haze.

The opitcal haze at wavelength λ is defined as:

(6)

$$H_{\lambda}={\tau_{T}(\lambda)}/{\tau_{s}(\lambda)}\times100\%$$

where $\tau_{T}$ is the total transmittance, and $\tau_{s}$ is the diffuse/scattering transmittance at λ.

The total transmittance in the visible region is calculate as:

(7)

$$\Gamma_{T}=\frac{\int_{380}^{780} \tau_{T}(\lambda)\cdot V(\lambda)\cdot S_{d65}\left( \lambda\right) d\lambda}{\int_{380}^{780} \tau_{T}(\lambda)\cdot S_{d65}\left( \lambda\right) d\lambda}\times100\%$$

The scattering transmittance in the visible region $\Gamma_{S}$ is calculate as:

(8)

$$\Gamma_{S}=\frac{\int_{380}^{780} \tau_{S}(\lambda)\cdot V(\lambda)\cdot S_{d65}\left( \lambda\right) d\lambda}{\int_{380}^{780} \tau_{S}(\lambda)\cdot S_{d65}\left( \lambda\right) d\lambda}\times100\%$$

where $\tau_{T}(\lambda)$ and $\tau_{S}(\lambda)$ are the total and diffuse transmittance at wavelength λ, respectively. $V(\lambda)$ is the CIE photopic spectral luminous efficiency function. $S_{d65}\left( \lambda\right)$is the relative spectral power distribution of CIE standard illuminant D65. $V(\lambda)$ and $S_{d65}\left( \lambda\right)$ can be obtained from Munsell Color Science Lab of RIT.

The haze in the visible region is defined as

(9)

$$H_{v}={\Gamma_{T}}/{\Gamma_{S}}\times100\%$$

**Supporting Figures**


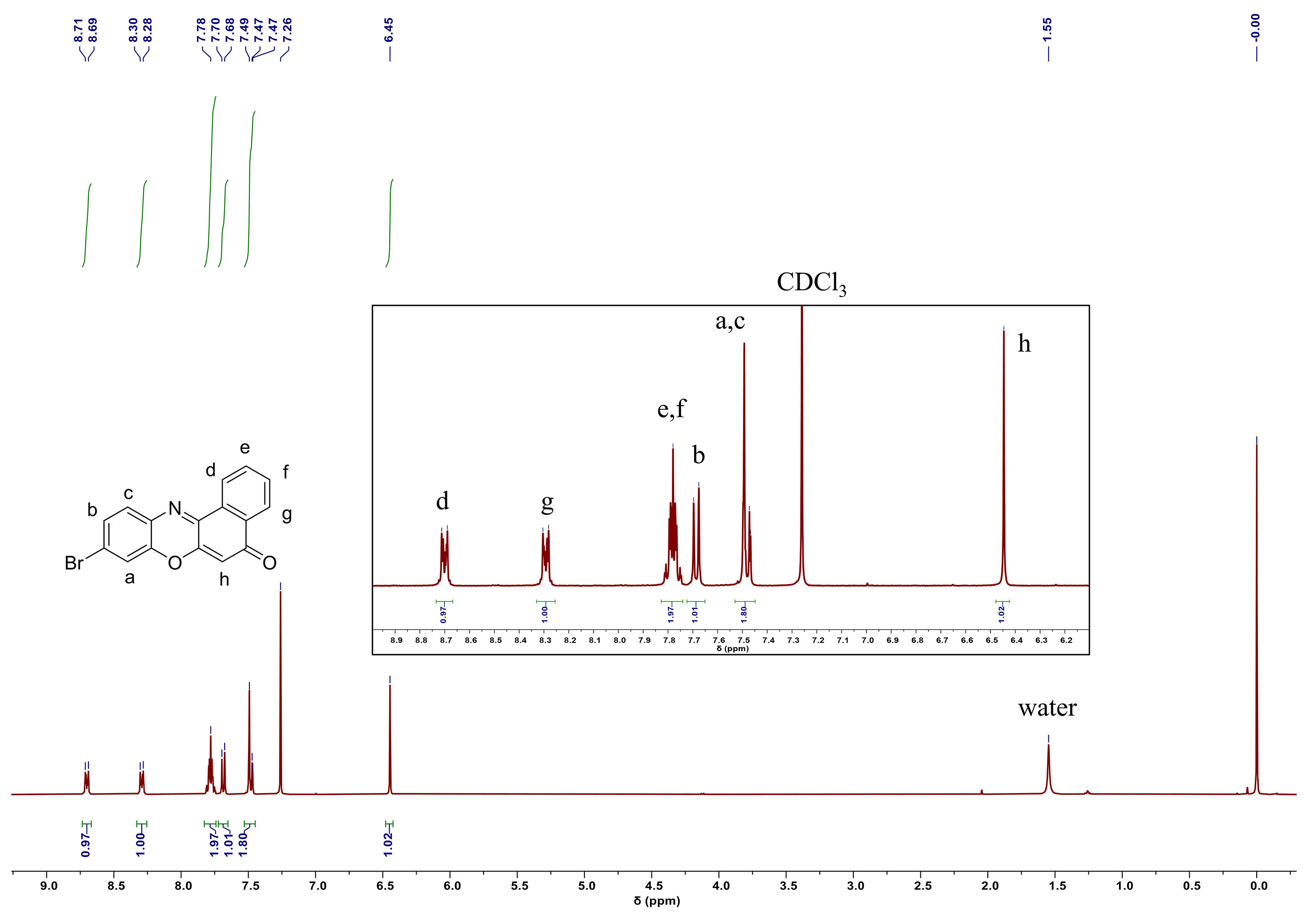


**Figure S2.** ^1^H NMR spectrum (400 MHz) of compound **1** in CDCl_3_.


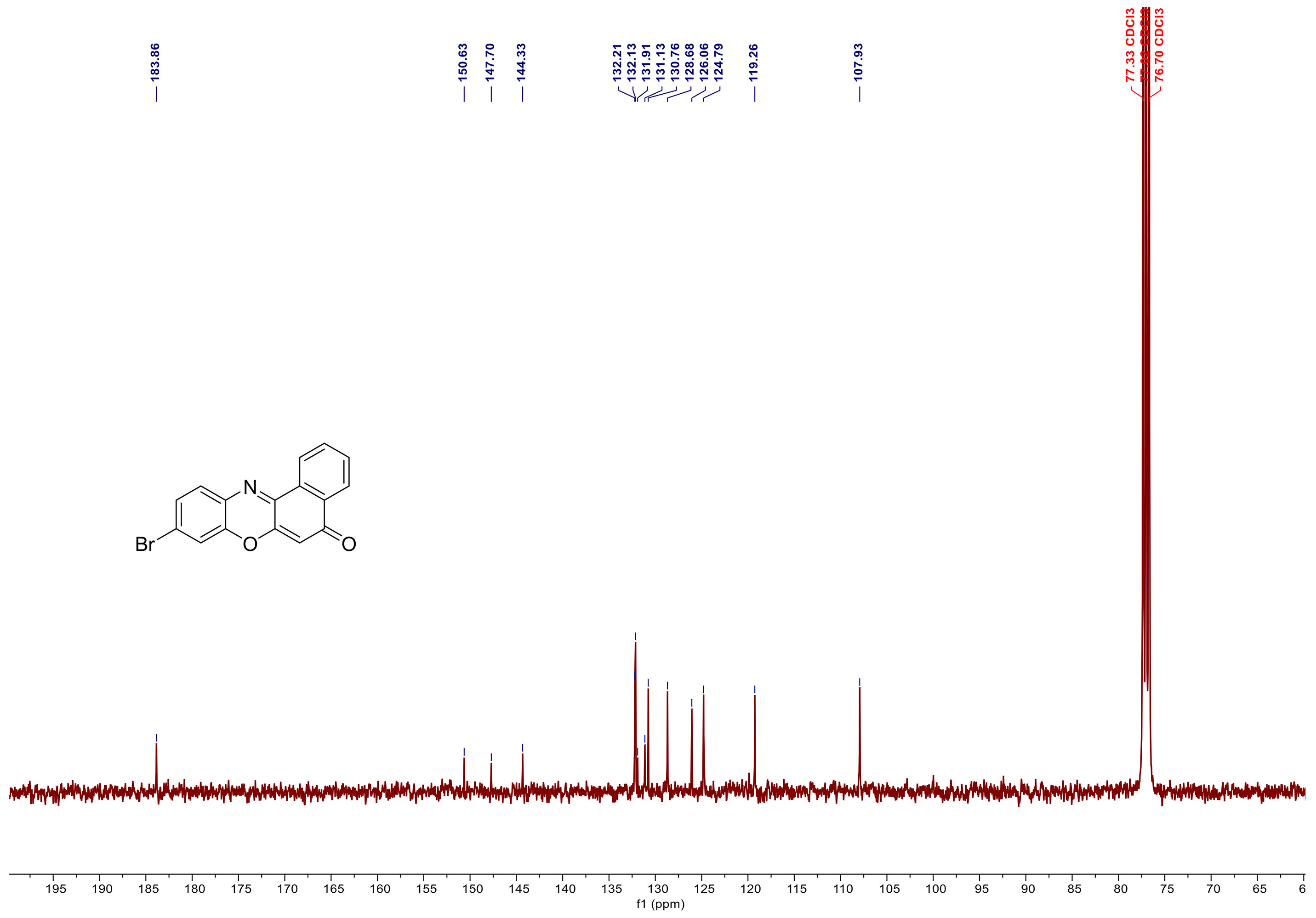


**Figure S3.** ^13^C NMR spectrum (100 MHz) of compound **1** in CDCl_3_.


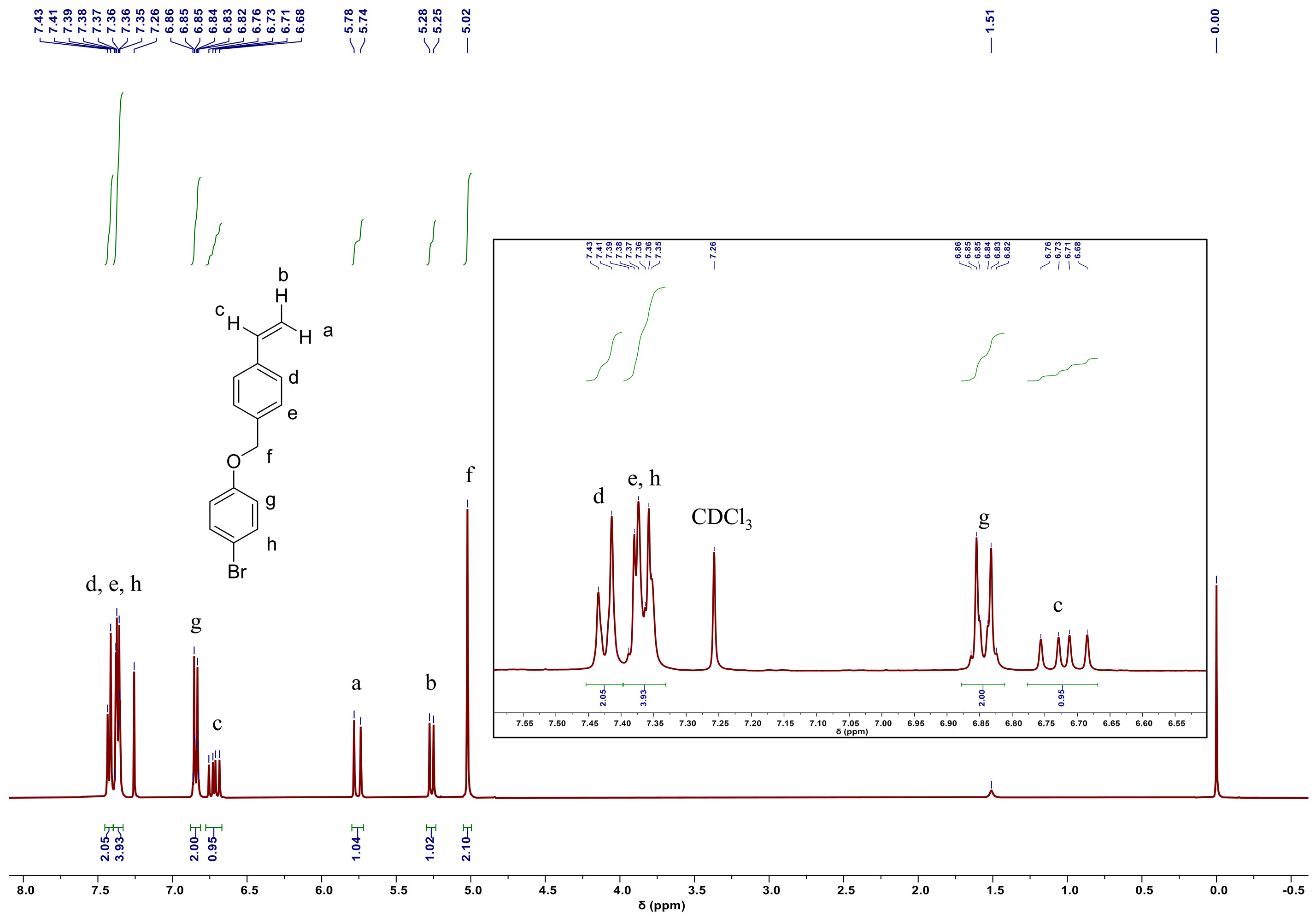


**Figure S4** ^1^H NMR spectrum (400 MHz) of compound **2** in CDCl_3_.


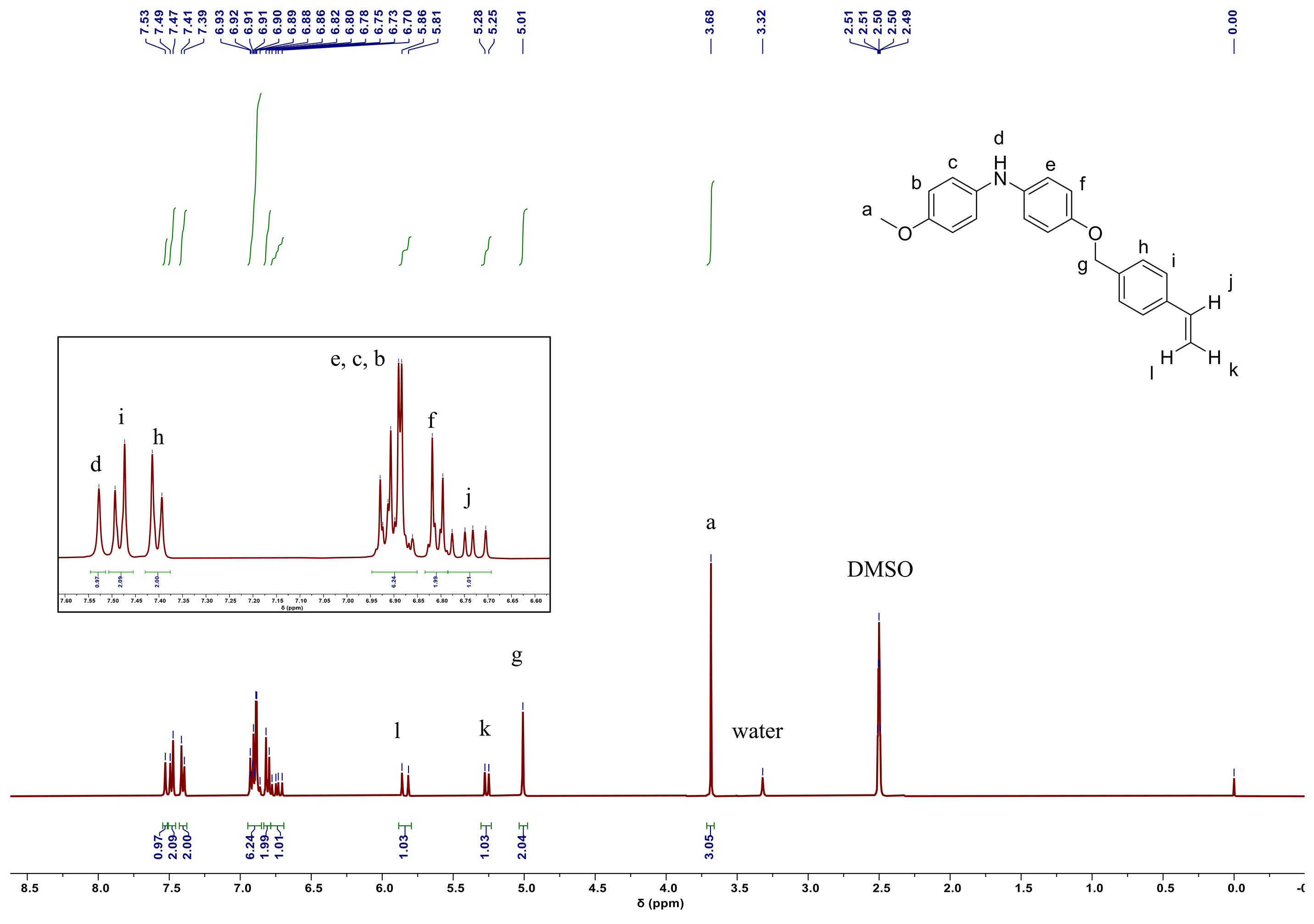


**Figure S5** ^1^H NMR spectrum (400 MHz) of compound **3** in DMSO-d_6_.


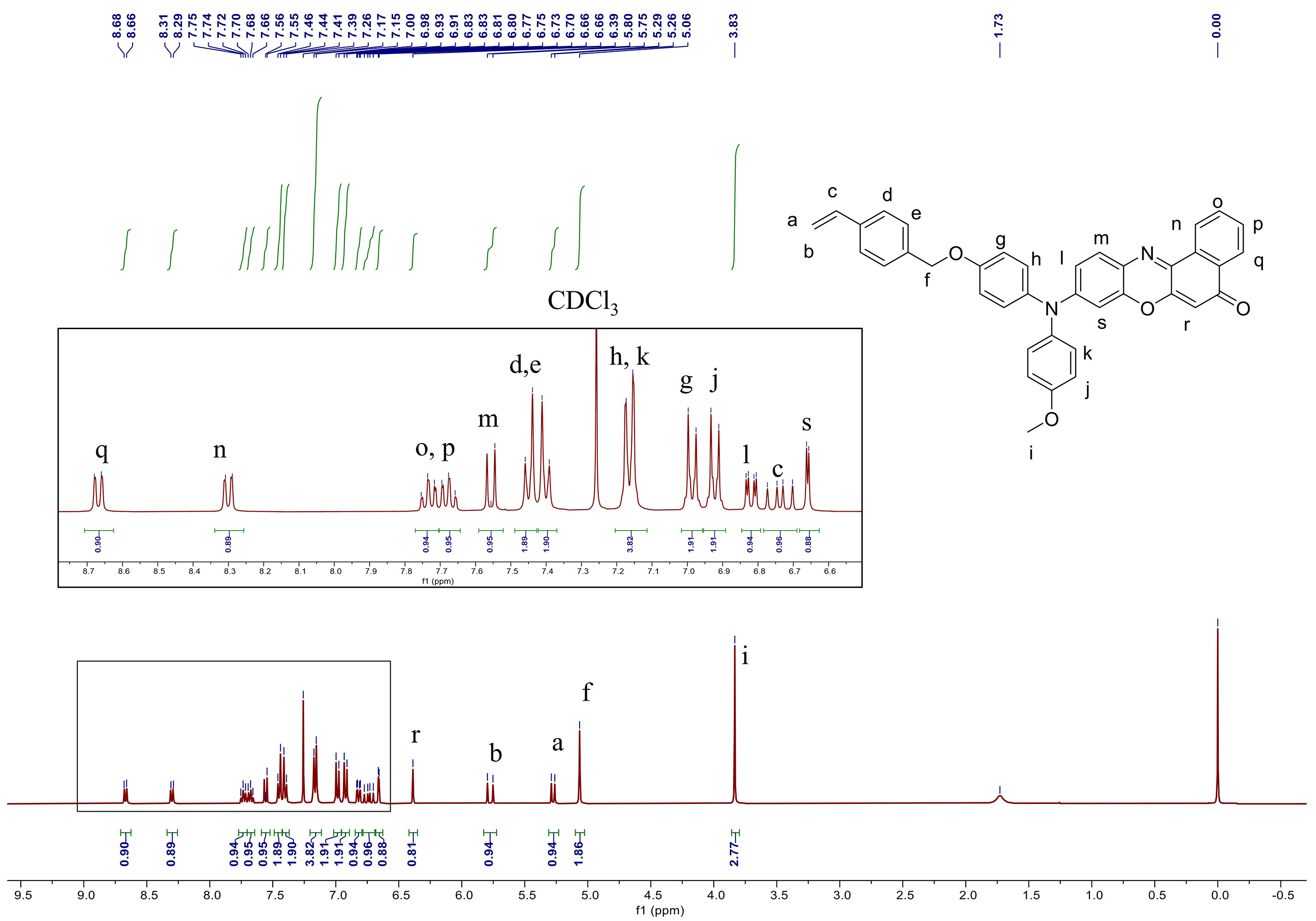


**Figure S6** ^1^H NMR spectrum (400 MHz) of Nile-DPA-VB in CDCl_3_.


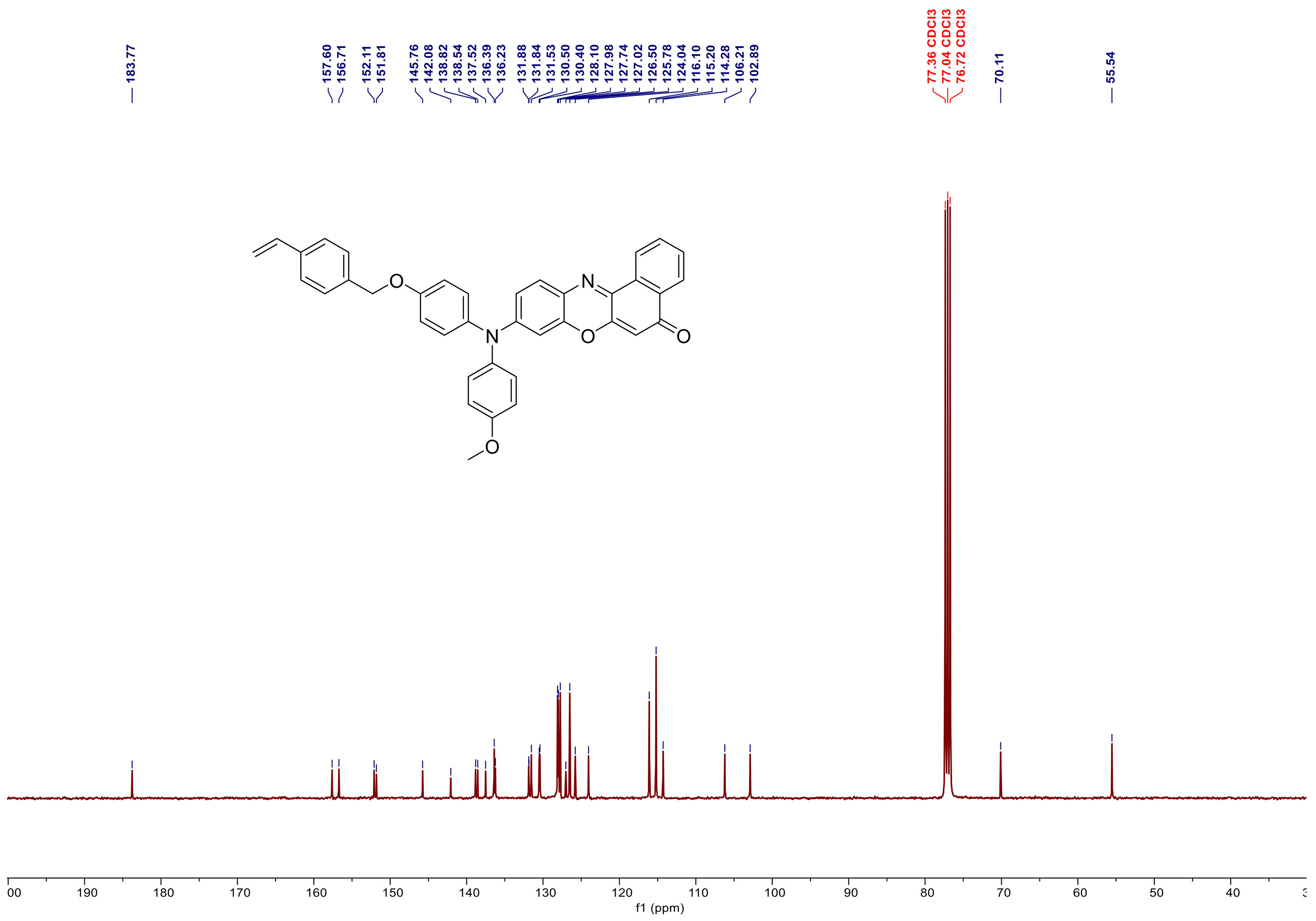


**Figure S7** ^13^C NMR spectrum (100 MHz) of Nile-DPA-VB in CDCl_3_.

**Figure S8** High resolution mass spectrum of Nile-DPA-VB.


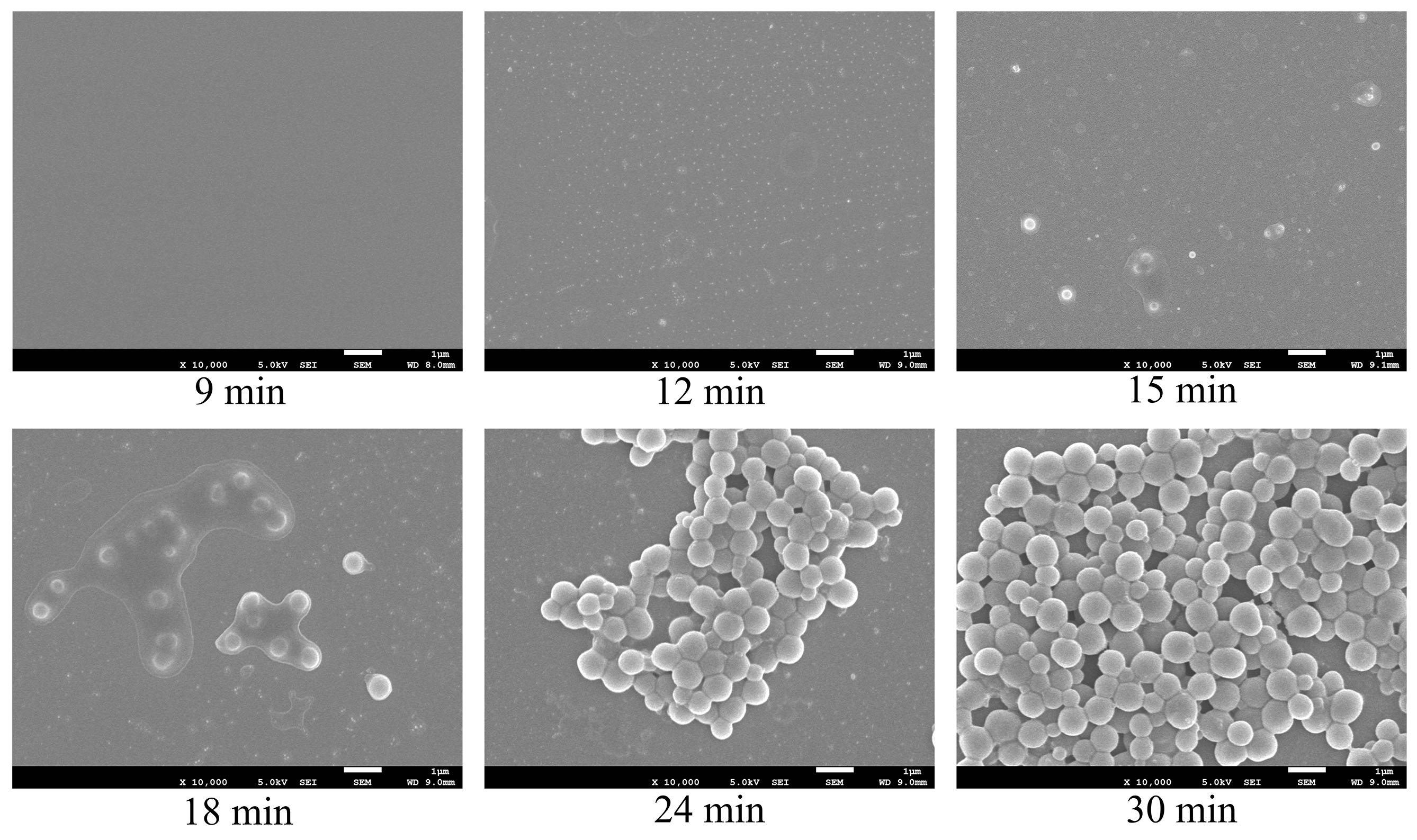


**Figure S9** SEM images of FPPs at different reaction time in the initial stage. Scale bar: 1 μm. Magnification: 10000.


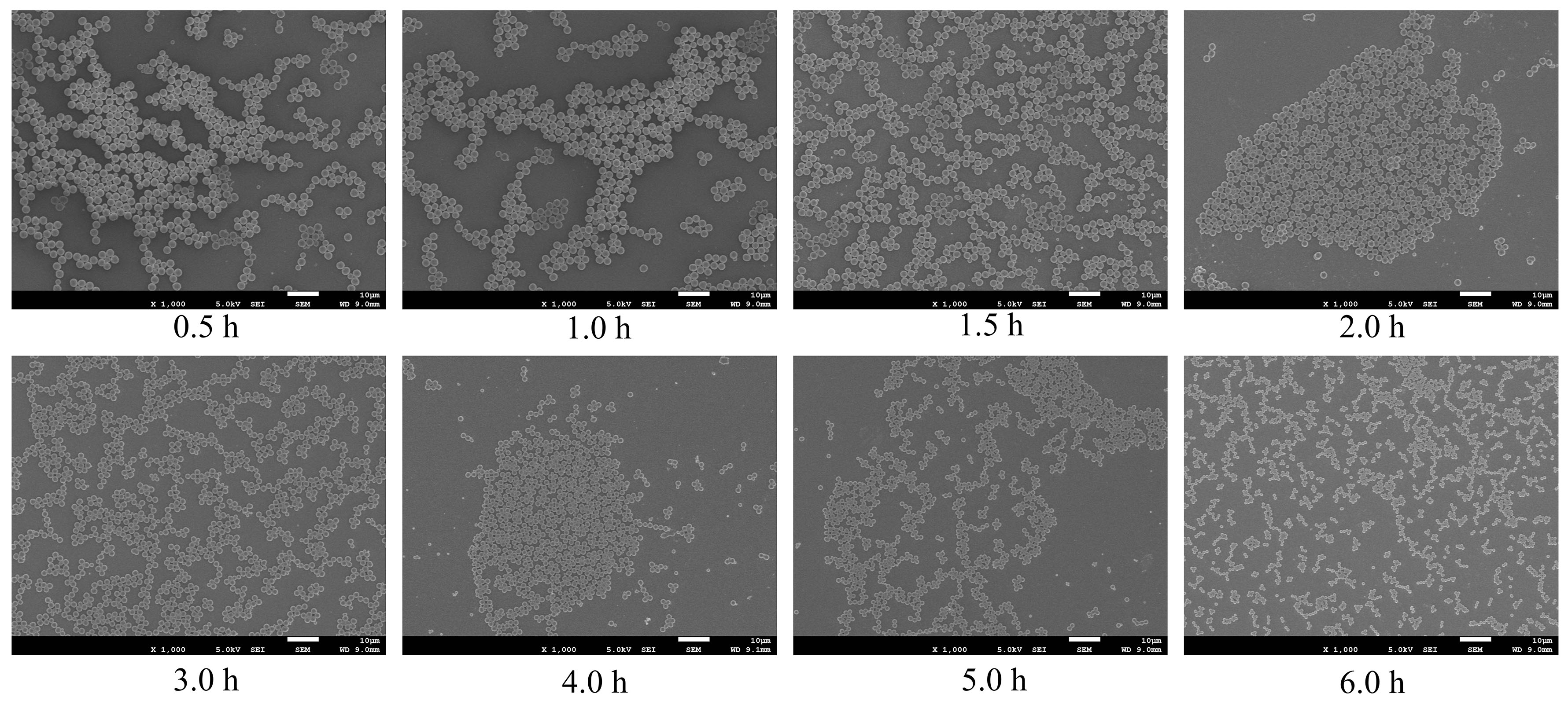


**Figure S10** SEM images of FPPs at different reaction time. Scale bar: 10 μm. Magnification: 1000.


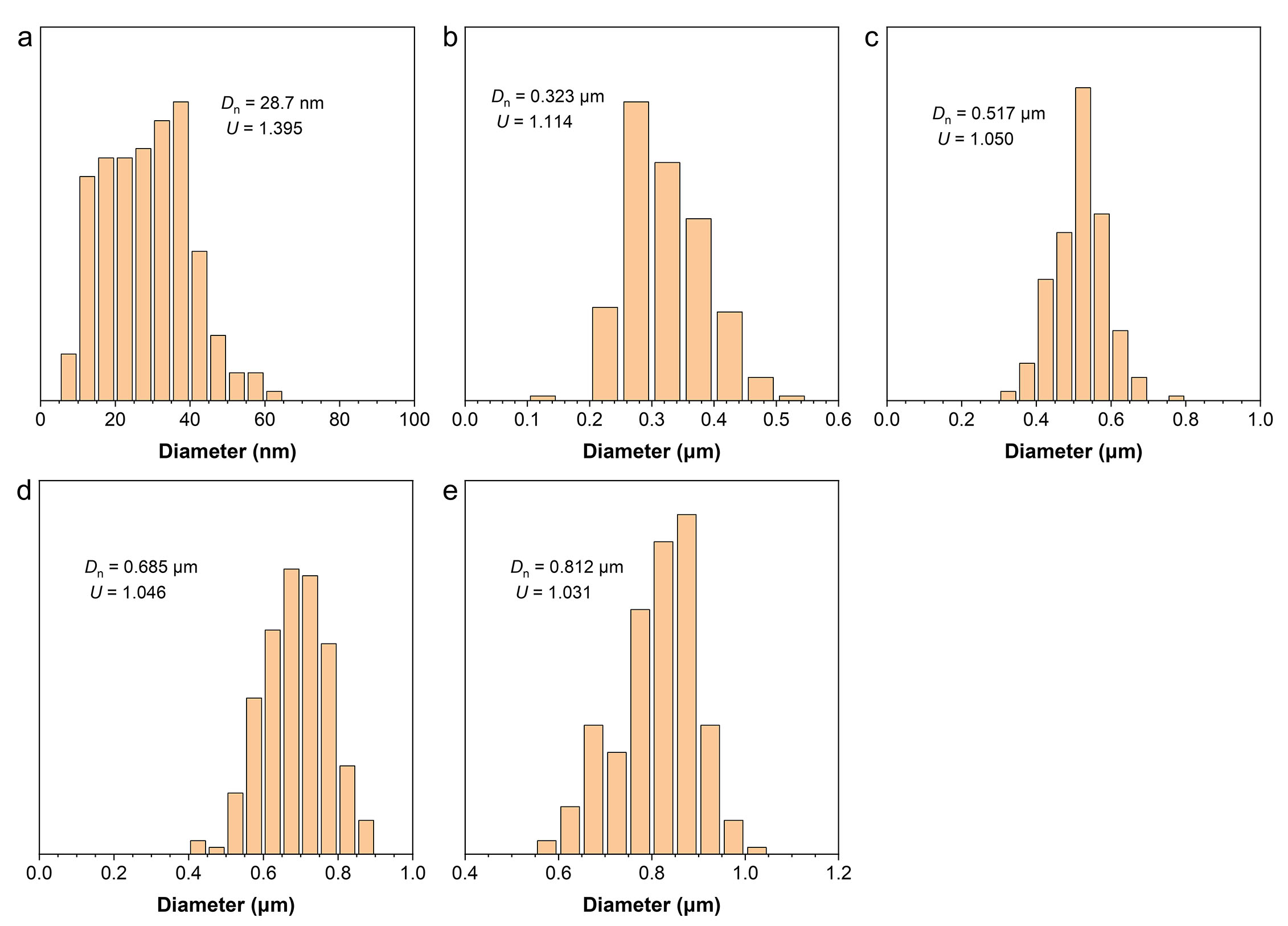


**Figure S11** Diameter distribution of FPPs. Reaction time at (a) 12 min, (b) 15 min, (c) 18 min, (d) 24 min, (e) 30 min.


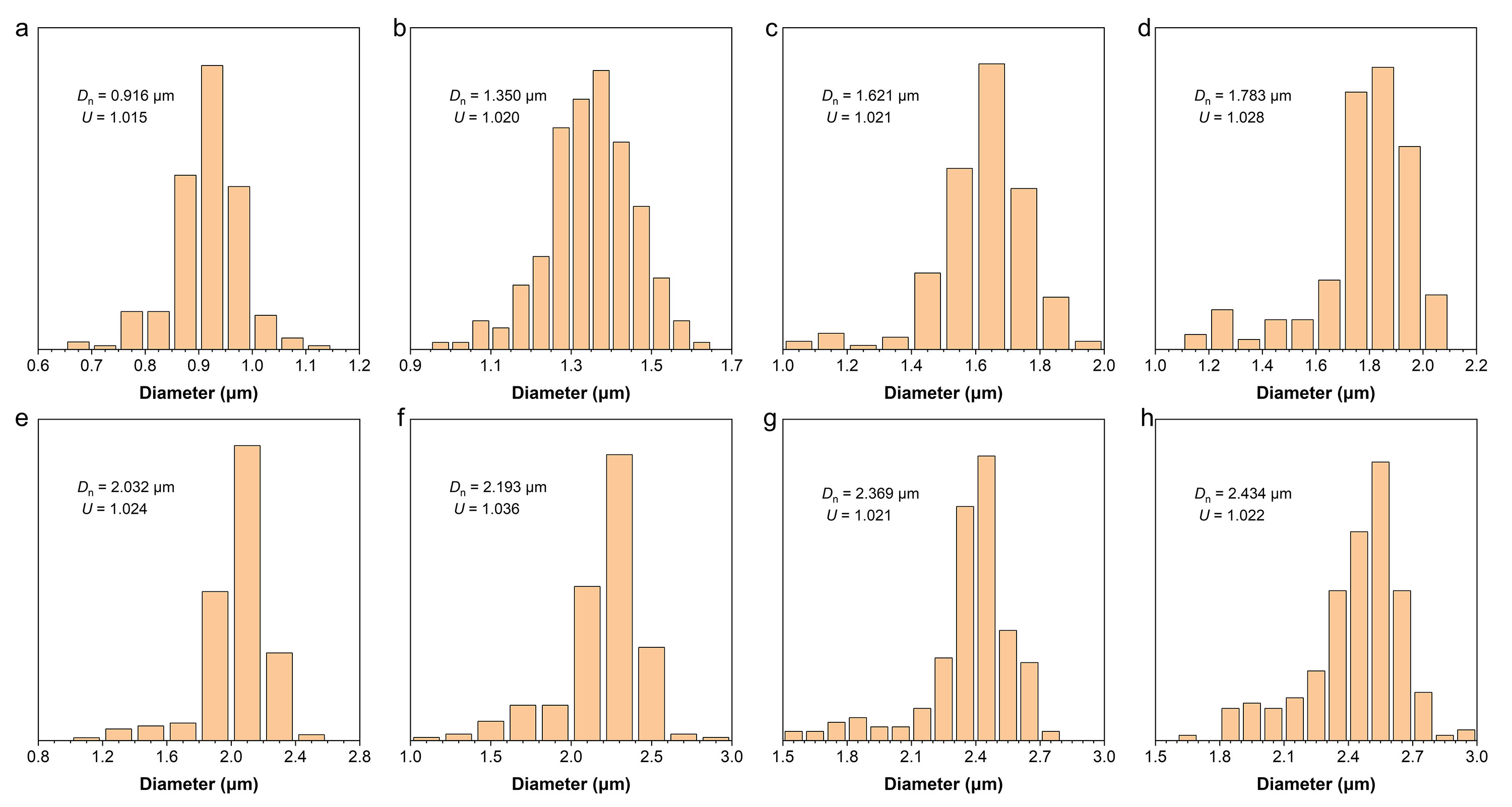


**Figure S12** Diameter distribution of FPPs. Reaction time at (a) 30 min, (b) 60 min, (c) 90 min, (d) 120 min, (e) 180 min, (f) 240 min, (g) 300 min, (h) 360 min.

**Figure S13** Average particle diameter change of FPPs.


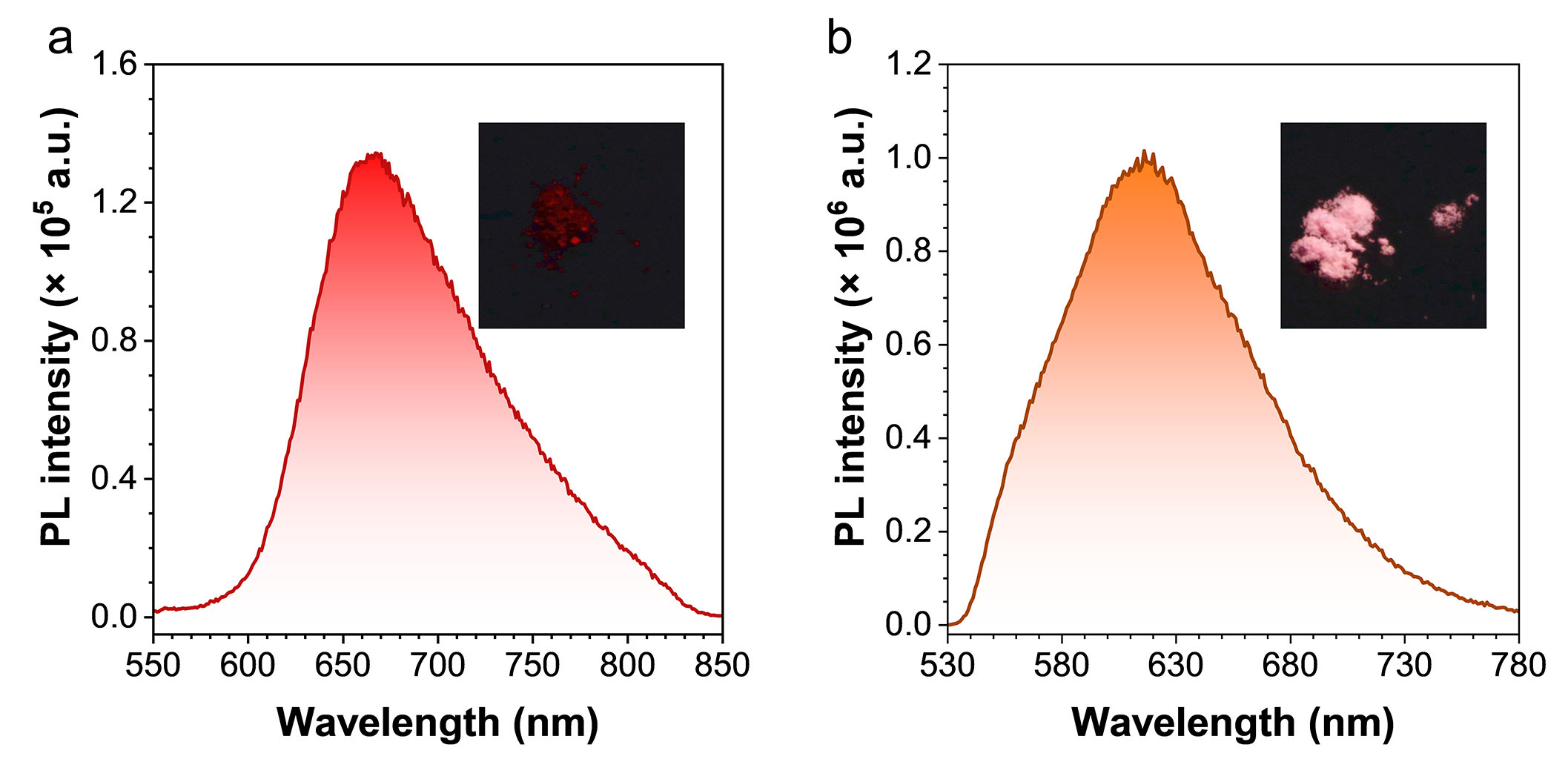


**Figure S14** PL spectrum of (a) Nile-DPA-VB powder, (b) FPPs powder at 6 h. $\lambda_{\mathrm{ex}}$ = 460 nm. Insets: Photographs of Nile-DPA-VB powder and FPPs powder 365 nm UV irradiation.


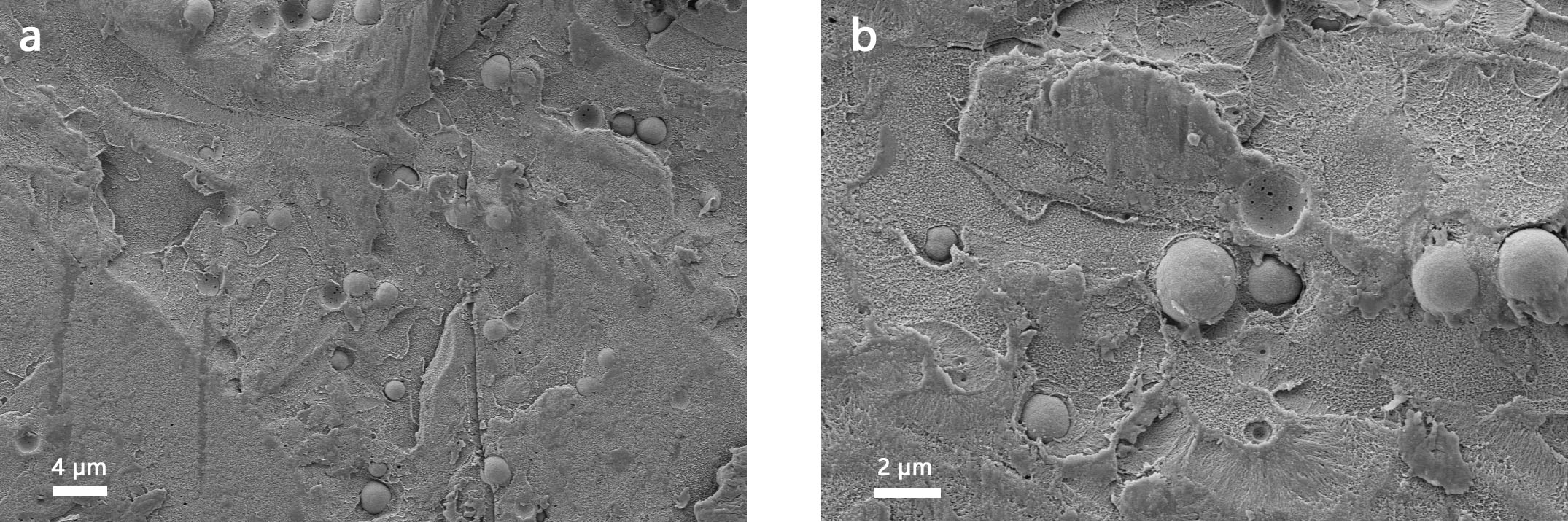


**Figure S15** Cross section SEM images of PMMA films doped with FPPs. The diameter of FPPs is 2.434 μm and the doping rate is 2.5%.


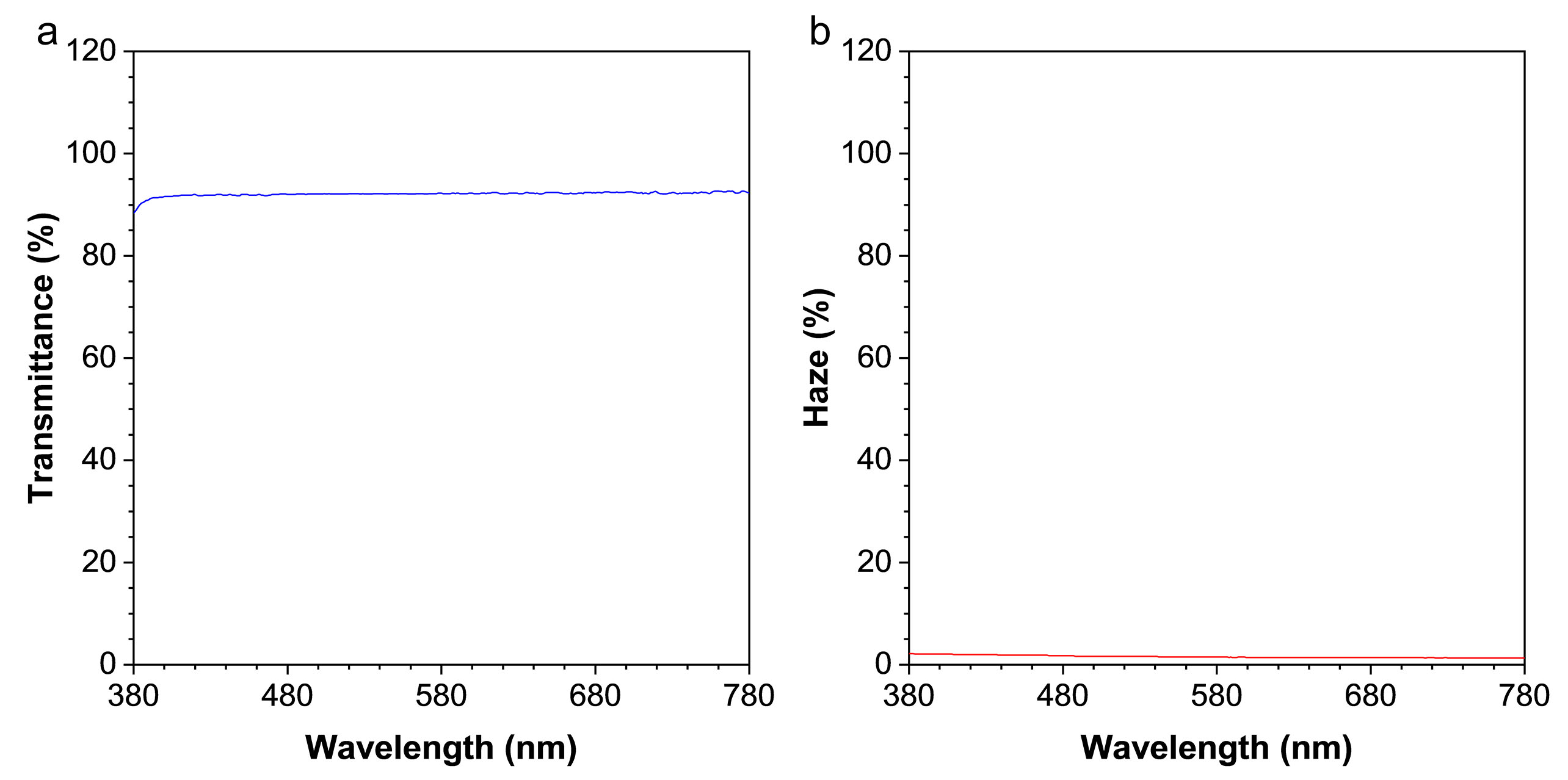


**Figure S16** (a) Transmittance and (b) haze spectrum of pure PMMA films.


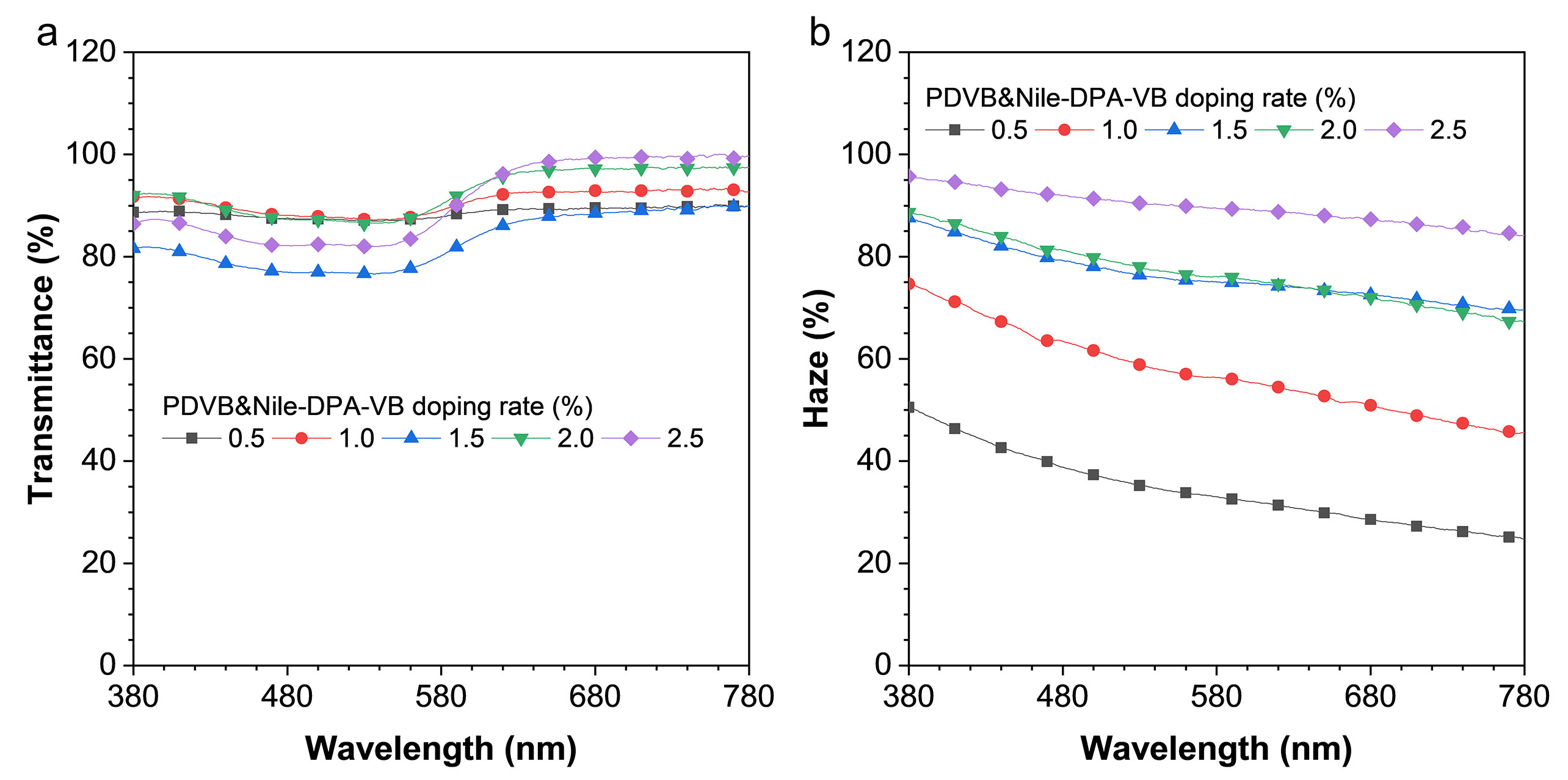


**Figure S17** (a) Transmittance and (b) haze spectrum of PMMA films doped with FPPs have a diameter of 0.916 μm.


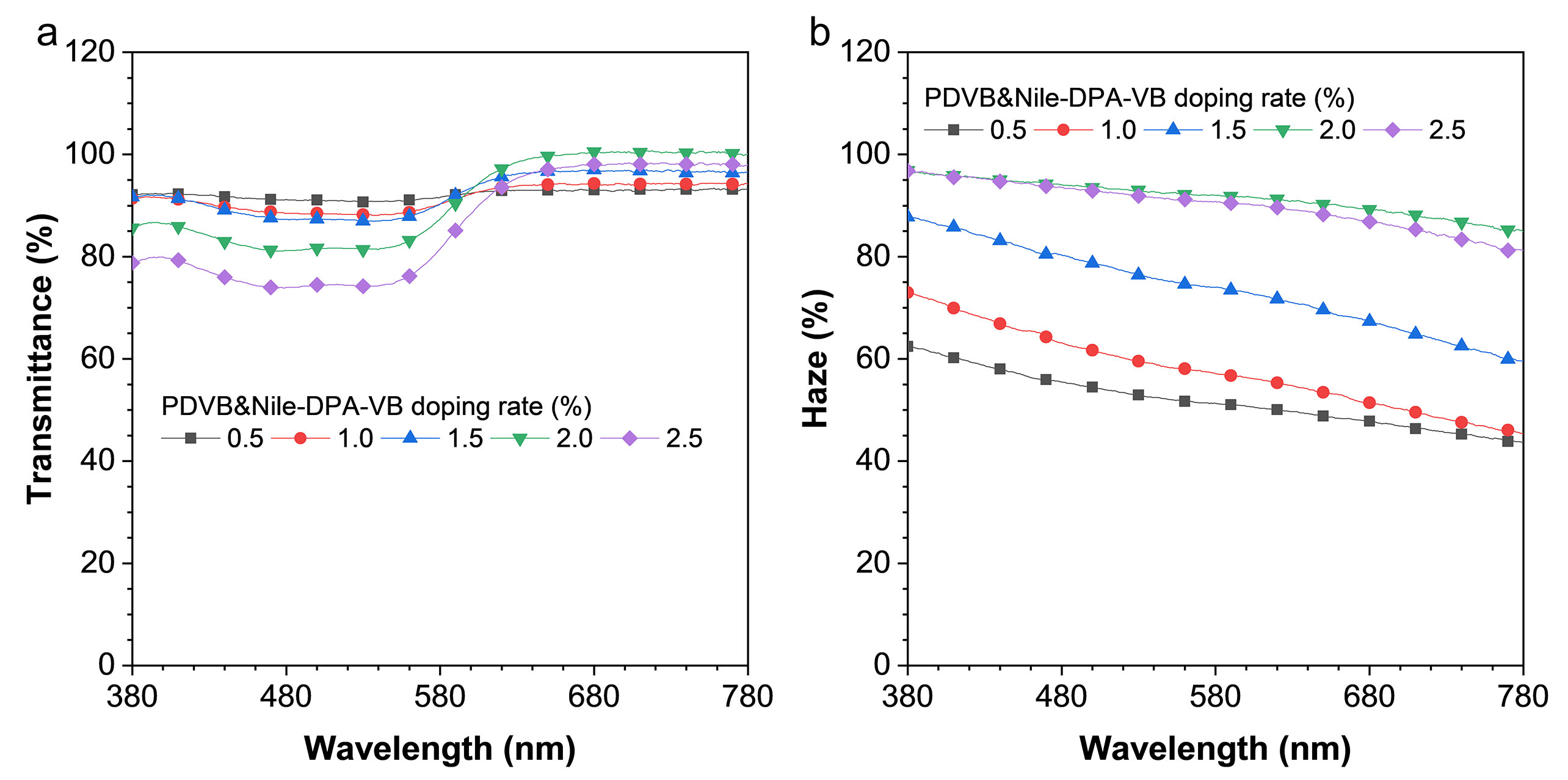


**Figure S18** (a) Transmittance and (b) haze spectrum of PMMA films doped with FPPs have a diameter of 1.350 μm.

**
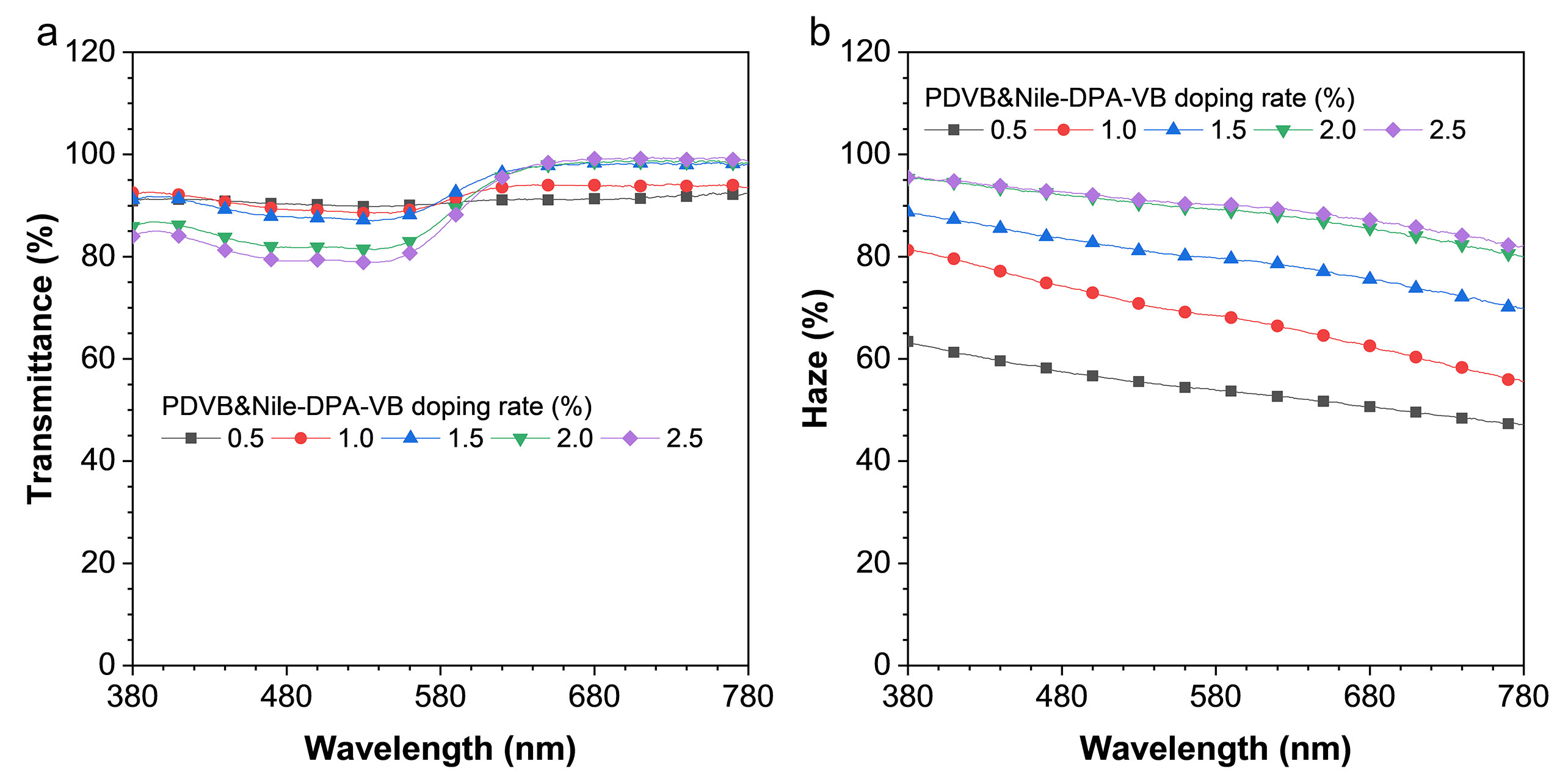
**

**Figure S19** (a) Transmittance and (b) haze spectrum of PMMA films doped with FPPs have a diameter of 1.783 μm.


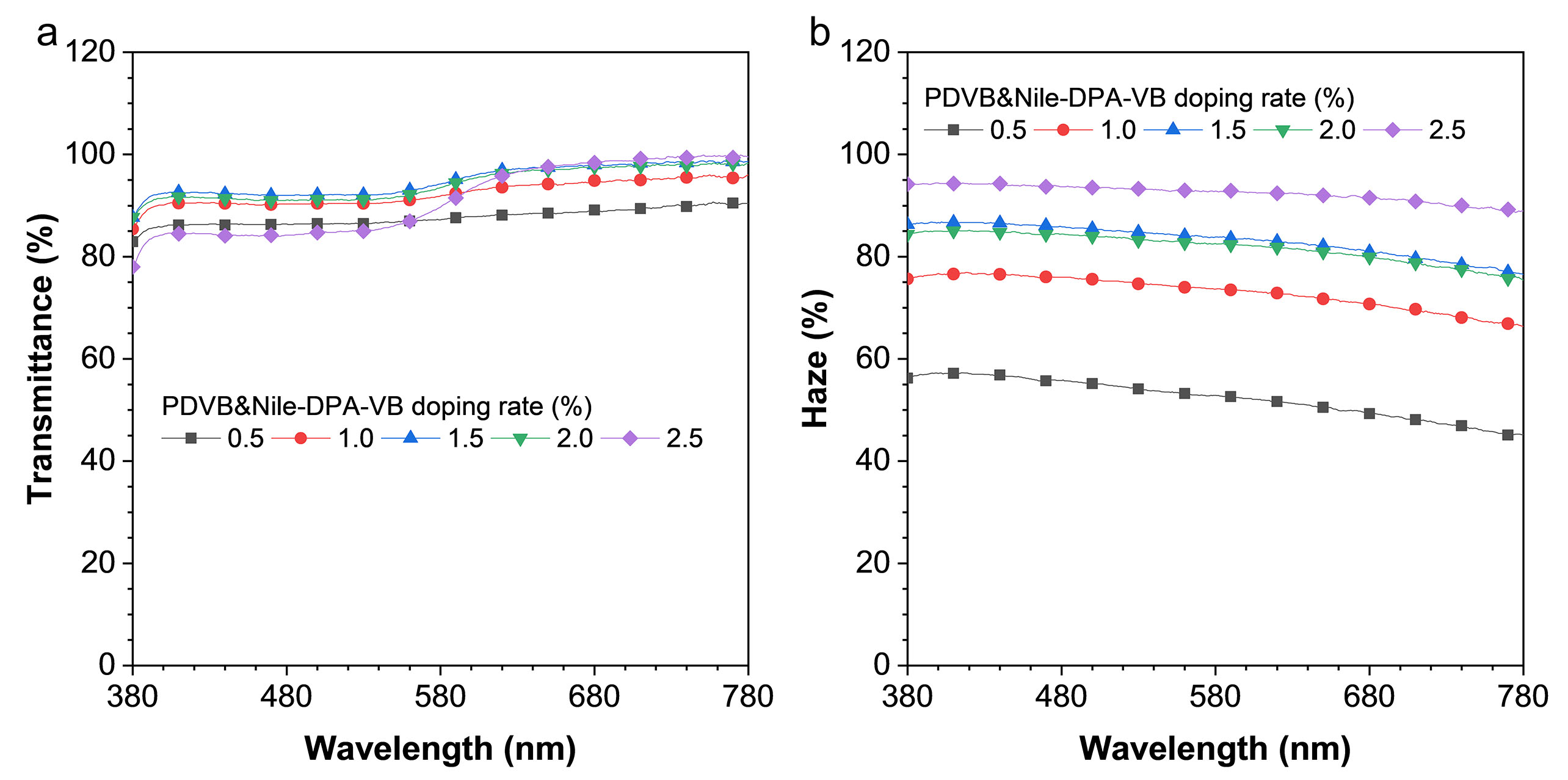


**Figure S20** (a) Transmittance and (b) haze spectrum of PMMA films doped with FPPs have a diameter of 2.193 μm.


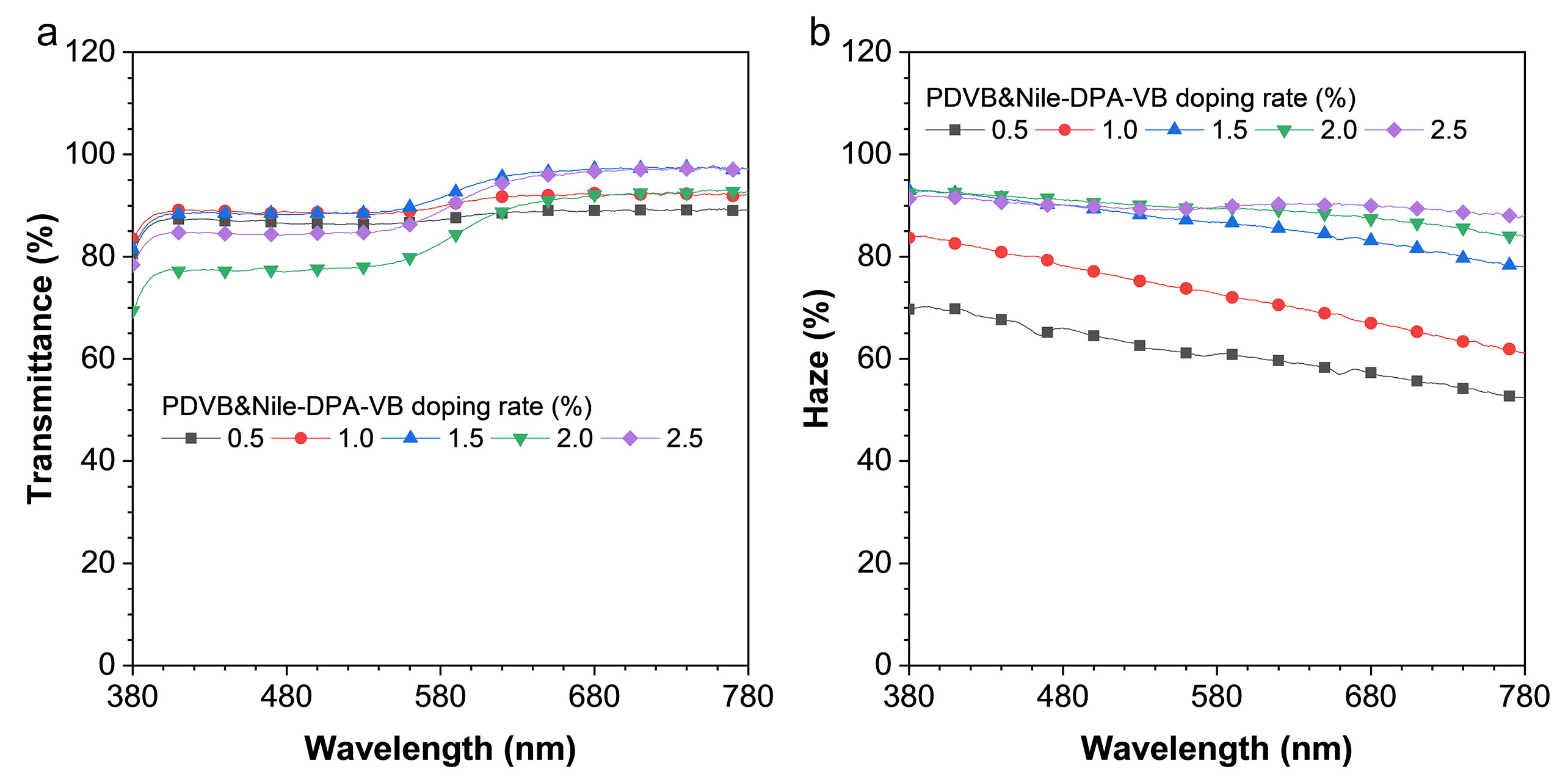


**Figure S21** (a) Transmittance and (b) haze spectrum of PMMA films doped with FPPs have a diameter of 2.434 μm.

**Reference**

[1] S. Abraham, S. Mangalath, D. Sasikumar, J. Joseph *Chem. Mater.*, **2017**, *29*, 9877-9881.

[2] J. Cao, Q.M. Liu, S.J. Bai, H.C. Wang, X. Ren, Y.X. Xu *ACS Appl. Mater. Interfaces*, **2019**, *11*, 29814-29820.

[3] J. Tan, G. Zhao, Y. Lu, Z. Zeng, M.A. Winnik *Macromolecules*, **2014**, *47*, 6856-6866.
